# Quantitative sensory testing and chronic pain syndromes: a cross-sectional study from TwinsUK

**DOI:** 10.1136/bmjopen-2024-085814

**Published:** 2024-09-03

**Authors:** Amber Rhee, Isabelle Granville Smith, Roger Compte, Jelle Vehof, Ayrun Nessa, Samuel Wadge, Maxim B Freidin, David L Bennett, Frances M K Williams

**Affiliations:** 1 Department of Twin Research and Genetic Epidemiology, King's College London, London, UK; 2 Department of Epidemiology, Johns Hopkins Bloomberg School of Public Health, Baltimore, Maryland, USA; 3 Departments of Ophthalmology and Epidemiology, University of Groningen, Groningen, The Netherlands; 4 Department of Ophthalmology, Vestfold Hospital Trust, Tønsberg, Norway; 5 Department of Biology, Queen Mary University of London, London, UK; 6 Nuffield Department of Clinical Neurosciences, Oxford University, Oxford, UK

**Keywords:** Chronic Pain, Cross-Sectional Studies, EPIDEMIOLOGIC STUDIES, Irritable Bowel Syndrome, Dry Eye Syndromes

## Abstract

**Abstract:**

**Objective:**

The chronic pain syndromes (CPS) include syndromes such as chronic widespread pain (CWP), dry eye disease (DED) and irritable bowel syndrome (IBS). Highly prevalent and lacking pathognomonic biomarkers, the CPS are known to cluster in individuals in part due to their genetic overlap, but patient diagnosis can be difficult. The success of quantitative sensory testing (QST) and inflammatory biomarkers as phenotyping tools in conditions such as painful neuropathies warrant their investigation in CPS. We aimed to examine whether individual QST modalities and candidate inflammatory markers were associated with CWP, DED or IBS in a large, highly phenotyped population sample.

**Design:**

Cross-sectional study.

**Setting:**

Community-dwelling cohort.

**Participants:**

Twins from the TwinsUK cohort

**Primary and secondary outcome measures:**

We compared 10 QST modalities, measured in participants with and without a CWP diagnosis between 2007 and 2012. We investigated whether inflammatory markers measured by Olink were associated with CWP, including interleukin-6 (IL-6), IL-8, IL-10, monocyte chemoattractant protein-1 and tumour necrosis factor. All analyses were repeated in DED and IBS with correction for multiple testing.

**Results:**

In N=3022 twins (95.8% women), no association was identified between individual QST modalities and CPS diagnoses (CWP, DED and IBS). Analyses of candidate inflammatory marker levels and CPS diagnoses in n=1368 twins also failed to meet statistical significance.

**Conclusion:**

Our findings in a large population cohort suggest a lack of true association between singular QST modalities or candidate inflammatory markers and CPS.

**STRENGTHS AND LIMITATIONS OF THIS STUDY**

- Large sample (N=3022) from a well-characterised, cohort.
- Appropriate correction for multiple testing across all analyses.
- Adjusted for major covariates (family relatedness, age, body mass index) in all analyses.
- Inclusion of participants with common painful conditions other than chronic pain syndromes in analysis.
- Variation in sample sizes for each quantitative sensory testing modality.

## Introduction

 Chronic pain is a major public health burden and leading cause of disability globally.^1^ Because of its clinical heterogeneity, the necessity of pain phenotyping and measurement tools are widely acknowledged.^2^ Chronic pain syndromes (CPS) are a recognised cluster of highly prevalent syndromes, including chronic widespread musculoskeletal pain (CWP)/fibromyalgia, dry eye disease (DED) and irritable bowel syndrome (IBS).^3^,^6^ CPS demonstrate genetic and symptomatology overlaps in the TwinsUK cohort, a large population-based cohort of community-dwelling twins, a finding supported in other population-based cohorts and clinical studies.^37^,^9^ Notably, CPS are characterised by lack of pathognomonic tissue injury or clinical biomarkers.^10^ Phenotyping and stratification, crucial steps to improving therapy success, thus prove difficult in these syndromes.

Quantitative sensory testing (QST) is a collection of psychophysical tests that assess percepts evoked by defined sensory stimuli.^11^ The tests evaluate a range of sensory modalities (ie, thermal and mechanical) in both the non-noxious and noxious range (ie, warm detection and heat pain thresholds (HPT)), providing insights into somatosensory nervous system functions.^12 13^ QST protocols have been standardised and are increasingly applied to clinical cohorts for grading neuropathic pain (pain caused by damage to the somatosensory nervous system) and subclassifying chronic pain.^14^,^18^ For instance, in neuropathic pain, QST can help build a sensory profile via an unbiased clustering algorithm, generating three principal groups: sensory loss, thermal hyperalgesia and mechanical hyperalgesia.^19^ Broadening the utilisation of QST beyond neuropathic pain is a subject of debate, however, as the extent of insight QST provides into the pathological mechanisms driving chronic pain is unclear.^20^,^23^

Inflammatory markers have been studied as potential phenotyping tools in chronic pain conditions. For example, osteoarthritis studies have identified biomarker profiles characteristic to the inflammatory osteoarthritis phenotype in synovial fluid.^24^ While CPS such as CWP were previously thought not inflammatory, some evidence suggests low-grade inflammation in CPS.^25^,^28^ Unlike QST, inflammatory markers offer direct insight into circulating mediators of disease state.^24^

This cross-sectional study aimed to examine the potential for QST and inflammatory markers to serve as phenotyping tools in CPS by investigating whether individual QST modalities and candidate inflammatory markers were associated with CPS diagnoses in a population-based cohort.

## Methods

### Participants

TwinsUK is an adult twin registry with over 15 000 volunteers, recruited from across the UK for research. Established in 1992, the ongoing longitudinal cohort, initially restricted to female recruitment, is predominantly white race/ethnicity and female (82%), with age mean 59.6 years (range 18–90+ years), and comprises monozygotic (MZ) and dizygotic (DZ) twins.^29^ TwinsUK has one of the largest QST datasets, administered to TwinsUK participants between 2007 and 2012 as part of collaborative studies with Pfizer.^30^ The cohort has been reported similar to age-matched British women from a singleton population-based cohort with regards to a range of health traits and diseases.^31^ Phenotypic data and biological specimens are collected from TwinsUK participants through annual questionnaires and approximately quadrennial clinic visits.^29^

Participants from TwinsUK were included in this cross-sectional study if they previously completed at least one QST measure and at least one CPS questionnaire. Participants were excluded from QST at the time of visit if they had severe skin disease, previous stroke or chemotherapy, likely impaired upper limb neurology, allergy to electrodes, history of melanoma, were pregnant or used painkillers on the day of the test.^30^

### Quantitative sensory testing protocols

In this study, we examined 10 QST modalities: cold intolerable threshold, cold pain threshold, heat pain supra threshold (HPST), HPT, mechanical detection threshold, mechanical pain threshold, skin flare extent, pain during burn induction, punctate hyperalgesia and thermal hyperalgesia. The last four modalities are part of a milder thermal burn protocol than the more commonly utilised protocol.^32^ QST was administered at a single site during standard TwinsUK visits. Protocols were established in TwinsUK in collaboration with the Stephen McMahon lab, King’s College London under the auspices of Prof D Bennett (coauthor, now at Oxford).^32^ Detailed descriptions of QST protocols are found in online supplemental file S1. A high degree of standardisation is necessary to perform QST accurately; to achieve this, nurses and research assistants underwent considerable training. Heritability and reliability of QST measures in this particular population have been formally assessed and reported previously, with heritability and inter-rater reliability estimates for each modality ranging from 0.29 to 0.55 and 0.34 to 0.91 respectively.^30 32^

### Candidate inflammatory marker measures

Five ‘candidate’ inflammatory markers were compiled *a priori* as exposure variables for secondary analysis: interleukin-6 (IL-6), IL-8, IL-10, monocyte chemoattractant protein-1 (MCP-1) and tumour necrosis factor (TNF). Markers were selected from the Olink Target 96 Inflammation panel to reflect both current literature and assay availability.^26^,^2833^ Serum inflammatory marker proteomics were collected and assayed as part of a large proteomics study. A subset of TwinsUK volunteers participated in both the QST and proteomics studies.

Olink uses Proximity Extension Assay to assess multiple proteins and report levels through a preprocessed relative (Normalised Protein eXpression) quantification on a log2 scale.^39 40^ Proteomics were assayed in two batches in 2019 and 2020, and data from the two batches were combined by author MBF into a single dataset through bridge sample normalisation according to Olink recommendations.^41 42^ Only samples collected within 2 years of participant QST visit were included in this study. For participants with multiple longitudinal samples, the sample collected on QST visit or closest to the participant’s QST visit was used in analysis.

### Chronic pain syndromes

CWP status was ascertained using a modified version of the London Fibromyalgia Epidemiology Study Screening Questionnaire.^43^ DED classification was determined according to the validated Women’s Health Study questionnaire, while IBS status was determined based on Rome 3 Criteria and, if unavailable, self-report of clinician diagnosis or treatment.^44^ Questionnaires were administered between 2002 and 2020. Participants were counted as cases if they were ever diagnosed with a CPS during this time.

### Statistical analysis

For each of the 10 QST modalities, we conducted a Mann-Whitney U test among participants who completed a CWP questionnaire; we compared scores between participants with CWP and participants without CWP (ie, comparing HPT between participants with CWP and participants without CWP). This was repeated for DED and IBS. With 10 QST modalities, a Bonferroni-correction cut-off was set at p=0.005.

To account for potential confounding due to CPS comorbidities, we conducted Mann-Whitney U tests in a sensitivity analysis comparing QST scores between participants with CWP and a control group of participants without any of the CPS diagnoses (‘true controls’). To address temporality, a secondary sensitivity analysis compared QST scores of participants with prevalent CWP diagnosis at QST date and participants with incident CWP diagnosis after QST date. In consideration of family relatedness and potential confounding by age and body mass index (BMI), we repeated the main analysis using mixed effects logistic regressions of each QST modality (scaled) on CWP diagnosis (ie, regression of HPST (scaled) on CWP diagnosis) with family ID as a random effect and age (scaled) and BMI category (nominal) as fixed effects. We used a BOBYQA (Bound Optimization BY Quadratic Approximation) optimisation technique, using the lme4 package in R.^45^ BMI categories were defined according to the Centers for Disease Control and Prevention BMI cut-off standards.^46^ All sensitivity analyses were repeated for DED and IBS.

For each of the five candidate inflammatory markers, we conducted a mixed effects logistic regression of inflammatory marker level on CWP diagnosis (ie, regression of IL-6 on CWP diagnosis) in a subset of participants who had Olink proteomics data collected within 2 years of their QST data collection visit. Model specifications were identical to those of the regression analyses in QST. Fixed effects included age (scaled) and BMI category (nominal). Family ID was included as a random effect to control for twin relatedness. Men were excluded from inflammatory panel analyses due to small sample size. This was repeated for DED and IBS. In total, we considered 15 models, examining five markers in each CPS, and imposed a Bonferroni-corrected p value cut-off of 0.003.

In a sensitivity analysis, we conducted a discordant twin analysis (MZ and DZ) of inflammatory marker levels on CWP diagnosis in twin pairs who were discordant for CWP. Using a conditional logistic regression analysis in the R survival package, associations were adjusted for BMI category (nominal).^47^ This analysis was restricted to samples collected on the same day as QST visit. We repeated these analyses in DED and IBS and imposed a Bonferroni-corrected p-value cut-off of 0.003.

We analysed all data using R V.4.2.1 (R Foundation for Statistical Computing, Vienna, Austria).

### Patient and public involvement

Patients or the public were not involved in the design, or conduct, or reporting or dissemination plans of our research.

## Results

Participants with CWP questionnaire data (N=2996) completed at least one QST modality (table 1). Prevalence of CWP was 22.4%; n=564 reported CWP at the time of QST visit and n=106 developed incident CWP after QST visit. More participants with CWP were classified as obese (26.9%) compared with those without CWP (16.9%).

**Table 1:** Characteristics of participants who completed a CWP questionnaire and QST

	Total* (N=2996)	Participants without CWP (n=2326)	Participants with CWP (n=670)
Zygosity			
Dizygotic (%)	1435 (47.9)	1078 (46.3)	357 (53.3)
Monozygotic (%)	1550 (51.7)	1239 (53.3)	311 (46.4)
Missing (%)	11 (0.4)	9 (0.4)	2 (0.3)
Sex			
Female (%)	2872 (95.9)	2220 (95.4)	652 (97.3)
Male (%)	124 (4.1)	106 (4.6)	18 (2.7)
Age (years)			
Mean (SD)	57.4 (12.2)	56.2 (12.7)	61.3 (9.3)
BMI category			
Underweight (%)	35 (1.2)	29 (1.2)	6 (0.9)
Healthy (%)	1326 (44.3)	1084 (46.6)	242 (36.1)
Overweight (%)	1063 (35.5)	821 (35.3)	242 (36.1)
Obese (%)	572 (19.1)	392 (16.9)	180 (26.9)

* Total participants in TwinsUK who have completed a CWP questionnaire and at least one QST modality.

BMI, body mass indexCWP, chronic widespread pain; QST, quantitative sensory testing

Among participants with DED questionnaire data, N=2583 completed at least one QST modality (table 2). With prevalence of DED at 28.8%, approximately half of the DED cases (n=358) were prevalent at QST visit; n=387 were incident and developed after QST visit.

**Table 2:** Characteristics of participants who completed a DED questionnaire and QST

	Total* (N=2583)	Participants without DED (n=1838)	Participants with DED (n=745)
Zygosity			
Dizygotic (%)	1234 (47.8)	890 (48.4)	344 (46.2)
Monozygotic (%)	1340 (51.9)	939 (51.1)	401 (53.8)
Missing (%)	9 (0.3)	9 (0.5)	0 (0.0)
Sex			
Female (%)	2485 (96.2)	1750 (95.2)	735 (98.7)
Male (%)	98 (3.8)	88 (4.8)	10 (1.3)
Age (years)			
Mean (SD)	58.3 (11.2)	57.6 (11.8)	60.2 (9.7)
BMI category			
Underweight (%)	30 (1.2)	21 (1.1)	9 (1.2)
Healthy (%)	1159 (44.9)	793 (43.1)	366 (49.1)
Overweight (%)	919 (35.6)	674 (36.7)	245 (32.9)
Obese (%)	475 (18.4)	350 (19.0)	125 (16.8)

* Total participants in TwinsUK who have completed a DED questionnaire and at least one QST modality.

BMI, body mass indexDED, dry eye disease; QST, quantitative sensory testing

Participants with IBS questionnaire data (N=2677) completed at least one QST modality (table 3). Prevalence of IBS was 26.2%, n=368 of which was prevalent at QST visit and n=334 of which was incident after QST visit.

**Table 3:** Characteristics of participants who completed an IBS questionnaire and QST

	Total* (N=2677)	Participants without IBS (n=1975)	Participants with IBS (n=702)
Zygosity			
Dizygotic (%)	1272 (47.5)	945 (47.8)	327 (46.6)
Monozygotic (%)	1397 (52.2)	1022 (51.7)	375 (53.4)
Missing (%)	8 (0.3)	8 (0.4)	0 (0.0)
Sex			
Female (%)	2580 (96.4)	1895 (95.9)	685 (97.6)
Male (%)	97 (3.6)	80 (4.1)	17 (2.4)
Age (years)			
Mean (SD)	58.0 (11.5)	58.2 (11.4)	57.4 (11.7)
BMI category			
Underweight (%)	30 (1.1)	23 (1.2)	7 (1.0)
Healthy (%)	1202 (44.9)	873 (44.2)	329 (46.9)
Overweight (%)	949 (35.5)	718 (36.4)	231 (32.9)
Obese (%)	496 (18.5)	361 (18.3)	135 (19.2)

* Total participants in TwinsUK who have completed an IBS questionnaire and at least one QST modality.

BMI, body mass indexIBS, irritable bowel syndrome; QST, quantitative sensory testing

In total, N=3022 unique participants were included across analyses. Within QST participants who completed all three CPS questionnaires (n=2502; 82.8%), n=1156 were true controls and never diagnosed with any CPS. Overlap of each analytical group and their CPS diagnoses are demonstrated in online supplemental figure S1.

Most participants completed only heat QST modalities for the Pfizer study (n=2633) with n=365 participants completing both heat and mechanical QST modalities. Sample sizes for each QST modality are available in table 4.

**Table 4:** QST sample sizes by modality and CPS analytical group

QST	CWP	DED	IBS	Total participants
CIT	100	95	94	100
CPT	133	127	126	133
Flare extent	101	99	98	101
HPST	2276	2066	2125	2298
HPT	2973	2561	2656	2999
MDT	380	360	357	380
MPT	386	364	362	386
Pain during burn induction	101	99	98	101
Punctate hyperalgesia	100	98	97	100
Thermal hyperalgesia	101	99	98	101

Each cell represents the number of participants who completed the corresponding QST and CPS questionnaire (ie, participants who completed the HPT test and CWP questionnaire=2973). The total participants’ column indicates the total unique participants across all analytical groups who have completed the corresponding QST test (ie, total participants who completed HPT test and any of the three CPS questionnaires=2999).

CIT, cold intolerable threshold; CPS, chronic pain syndrome; CPT, cold painful threshold; CWP, chronic widespread pain; DED, dry eye disease; HPST, heat pain supra threshold; HPT, heat pain threshold; IBS, irritable bowel syndrome; MDT, mechanical detection threshold; MPT, mechanical painful thresholdQST, quantitative sensory testing

Of QST participants with CWP questionnaire data, N=1342 had data for inflammatory markers collected within 2 years of QST visit after excluding men (n=18) and analysed in mixed effects logistic regressions (online supplemental table S1). Prevalence of CWP was 27.5%; n=117 twin pairs discordant for CWP diagnosis had inflammatory markers collected on QST visit and were examined in the sensitivity analysis (online supplemental table S2).

Of QST participants with DED questionnaire data, N=1211 had data for inflammatory markers collected within 2 years of QST visit after excluding men (n=16) and analysed in mixed effects logistic regressions (online supplemental table S3). Consistent with the main QST sample, prevalence of DED was 30.4% at sample collection. There were n=129 twin pairs discordant for DED diagnosis who had inflammatory markers collected on QST visit and included in the sensitivity analysis (online supplemental table S4).

Of QST participants with IBS questionnaire data, N=1248 had data for inflammatory markers collected within 2 years of QST visit after excluding men (n=15) and analysed in the mixed effects logistic regressions (online supplemental table S5). Prevalence of IBS was 27.0%, similar to the main QST sample. There were n=125 twin pairs discordant for IBS diagnosis with inflammatory markers collected on QST visit who were examined in the sensitivity analysis (online supplemental table S6).

In total, N=1368 unique participants had inflammatory marker data across analyses. Overlap of each analytical group and their CPS diagnoses are displayed in online supplemental figure S2. All inflammatory marker samples were above limit of detection (LOD) for IL-10, MCP-1 and TNF with n=106 samples below LOD for IL-6 and n=9 samples below LOD for IL-8. Many participants (n=1230; 89.9%) had data for inflammatory marker levels collected within a year of their QST visit, with most (n=1147; 83.8%) samples obtained on the day of QST visit.

A flowchart of all study populations is documented in online supplemental figure S3.

### QST measures in CPS

**Figure 1:**
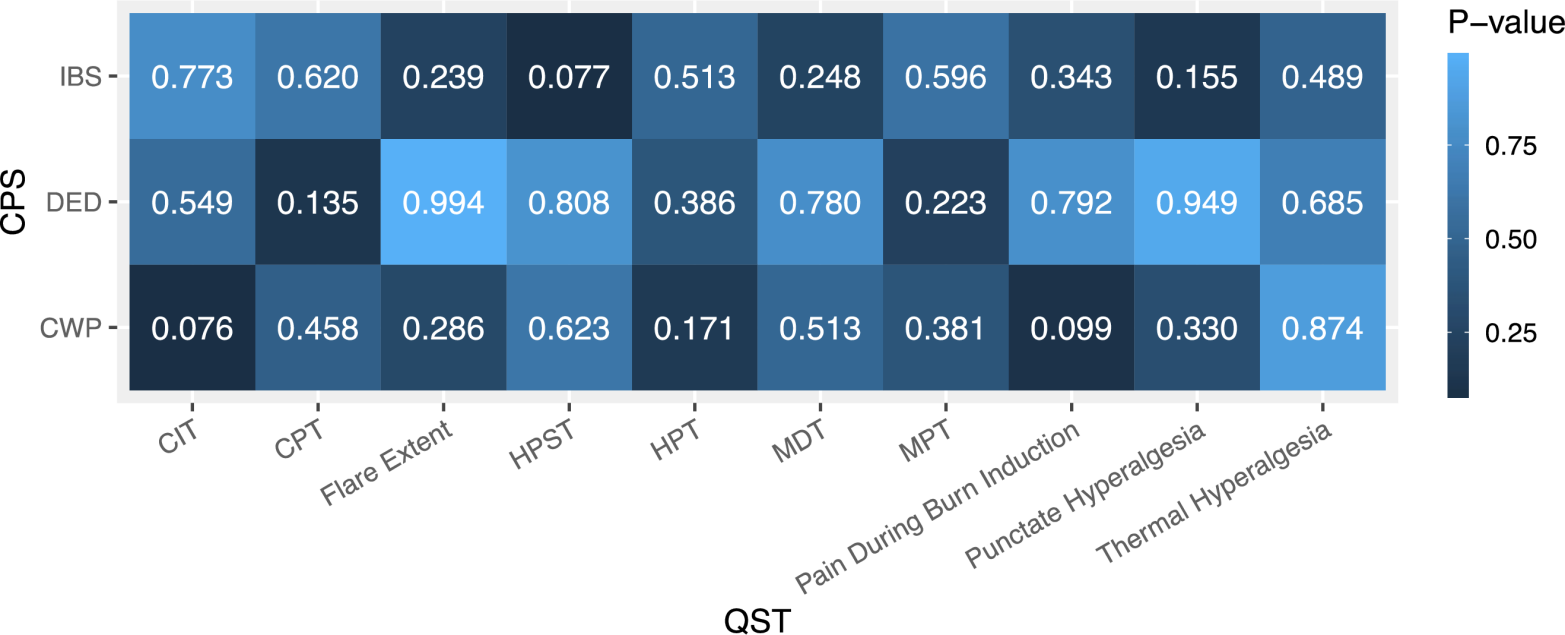
Heatmap of p values from Mann-Whitney U tests comparing QST scores in participants with and without CWP, DED and IBS. Each cell represents the p value of an individual Mann-Whitney U test for the corresponding QST in the relevant CPS questionnaire population (ie, p value for Mann-Whitney U test comparing CIT scores in participants with CWP and participants without CWP=0.076). Bonferroni-corrected p value threshold=0.005. CIT, cold intolerable threshold; CPT, cold pain threshold; CWP, chronic widespread pain; DED, dry eye disease; HPST, heat pain supra threshold; HPT, heat pain threshold; IBS, irritable bowel syndrome; MDT, mechanical detection threshold; MPT, mechanical pain threshold; QST, quantitative sensory testing.

We found no differences between the central tendencies of QST scores in participants with and without CWP for all 10 QST modalities (figure 1). Mann-Whitney U test p values ranged from 0.076 to 0.874 with a Bonferroni threshold of p=0.005. This finding was repeated in analyses comparing QST scores in participants with and without DED and in participants with and without IBS. Mann-Whitney U test p values in these CPS ranged from 0.135 to 0.994 and 0.077 to 0.773, respectively. Minimal detectable effect sizes (Cohen’s d) with 80.0% power for each test are found in online supplemental table S7.^48^ Of the total 30 Mann-Whitney U tests, comparison groups did not meet the unequal variances assumption in nine tests (online supplemental table S7). Thus, further inference about differences between medians in these tests cannot be made.

Sensitivity analyses comparing QST scores of participants with CWP and true controls were consistent with main analyses and not statistically significant (online supplemental figure S4). Comparisons of QST scores in participants with prevalent CWP and participants with incident CWP were also not statistically significant (online supplemental figure S5). Mixed effects regression analyses of QST on CWP, adjusted for twin relatedness, age (scaled) and BMI category (nominal), were also consistent with the main Mann-Whitney U findings and failed to reach statistical significance (online supplemental table S8). These findings were repeated in DED and IBS analyses (online supplemental figures S4 and S5, online supplemental table S8).

### Inflammation markers in CPS

In the CWP case–control mixed effects logistic regressions of inflammatory marker levels, no association reached statistical significance after Bonferroni-correction at p=0.003 (table 5). The association between IL-6 and CWP was nominally significant in a univariate model with an OR of 1.31 (95% CI 1.03 to 1.66) and p value of 0.030. All associations, however, were null after adjusting for age (scaled), BMI category (nominal) and twin relatedness.

**Table 5:** Mixed effects logistic regressions of inflammatory marker level on CWP diagnosis

Inflammatory marker level (NPX)	Univariate model*		Multivariate model†	
	OR (95% CI)	P value	OR (95% CI)	P value
IL-6	1.31 (1.03 to 1.66)	0.030	1.06 (0.82 to 1.38)	0.652
IL-8	1.28 (0.99 to 1.65)	0.055	1.17 (0.90 to 1.51)	0.237
IL-10	0.92 (0.69 to 1.22)	0.563	0.86 (0.64 to 1.15)	0.305
MCP-1	1.05 (0.76 to 1.47)	0.759	0.89 (0.63 to 1.25)	0.488
TNF	1.11 (0.82 to 1.50)	0.509	0.94 (0.68 to 1.29)	0.692

Bonferroni-corrected p- value threshold=0.003.

* Random effect: family ID; fixed effects: none.

† Random effect: family ID; fixed effects: age (scaled), BMI category (nominal).

BMIbody mass indexCWP, chronic widespread pain; IL-6, interleukin-6; IL-8, interleukin-8; IL-10, interleukin-10; MCP-1, monocyte chemoattractant protein-1; NPX, Normalised Protein eXpression; TNF, tumour necrosis factor

We found no association between any inflammatory marker and DED diagnosis in mixed effects logistic regressions (table 6). ORs for all inflammatory markers approximated 1.00 with p values ranging from 0.411 to 0.775.

**Table 6:** Mixed effects logistic regressions of inflammatory marker level on DED diagnosis

Inflammatory marker level (NPX)	Univariate model*		Multivariate model†	
	OR (95% CI)	P value	OR (95% CI)	P value
IL-6	1.11 (0.90 to 1.37)	0.336	1.07 (0.85 to 1.34)	0.584
IL-8	1.03 (0.82 to 1.28)	0.815	0.97 (0.77 to 1.22)	0.775
IL-10	1.08 (0.85 to 1.37)	0.528	1.06 (0.83 to 1.35)	0.654
MCP-1	1.15 (0.86 to 1.54)	0.342	1.06 (0.78 to 1.44)	0.701
TNF	1.21 (0.93 to 1.57)	0.146	1.12 (0.85 to 1.47)	0.411

Bonferroni-corrected p- value threshold=0.003.

* Random effect: family ID; fixed effects: none.

† Random effect: family ID; fixed effects: age (scaled), BMI category (nominal).

BMIbody mass indexDED, dry eye disease; IL-6, interleukin-6; IL-8, interleukin-8; IL-10, interleukin-10; MCP-1, monocyte chemoattractant protein-1; NPX, Normalised Protein eXpression; TNF, tumour necrosis factor

In the mixed effects logistic regressions of inflammatory marker levels on IBS diagnosis, the association between IL-8 and IBS diagnosis was nominally significant with an OR of 1.29 (95% CI 1.02 to 1.64) and p value of 0.036 (table 7). With a p value threshold of p=0.003, no association reached statistical significance with all other p values ranging from 0.249 to 0.893.

**Table 7:** Mixed effects logistic regressions of inflammatory marker level on IBS diagnosis

Inflammatory marker level (NPX)	Univariate model*		Multivariate model†	
	OR (95% CI)	P value	OR (95% CI)	P value
IL-6	0.94 (0.74 to 1.18)	0.580	0.98 (0.77 to 1.26)	0.893
IL-8	1.20 (0.95 to 1.52)	0.124	1.29 (1.02 to 1.64)	0.036
IL-10	1.13 (0.88 to 1.45)	0.349	1.15 (0.89 to 1.48)	0.283
MCP-1	1.00 (0.74 to 1.35)	0.988	1.11 (0.81 to 1.51)	0.522
TNF	1.07 (0.81 to 1.42)	0.632	1.18 (0.89 to 1.58)	0.249

Bonferroni-corrected p- value threshold=0.003.

* Random effect: family ID; fixed effects: none.

† Random effect: family ID; fixed effects: age (scaled), BMI category (nominal).

BMIbody mass indexIBS, irritable bowel syndrome; IL-6, interleukin-6; IL-8, interleukin-8; IL-10, interleukin-10; MCP-1, monocyte chemoattractant protein-1; NPX, Normalised Protein eXpression; TNF, tumour necrosis factor

In the discordant twin sensitivity analyses, no associations were detected between intrapair differences in inflammatory marker level and CWP, in agreement with the main analyses (Bonferroni correction at p=0.003; online supplemental table S9). These findings were also repeated in DED and IBS.

Full results of inflammatory marker mixed effects regression analyses are found in online supplemental table S10. Discordant twin analyses can be found in online supplemental table S11.

## Discussion

### Considerations for QST interpretation in CPS

This study is the first large-scale investigation of the association of individual QST modalities with CPS in a population-based cohort. With high heterogeneity in both presentation of pain and response to common treatments, identifying a patient’s pain phenotype, and optimal treatment, is a necessary next step to improving clinical care.^2^ QST is an appealing tool to assist in this endeavour because it is a semi-objective, quantitative method to potentially characterise pain.^49^

Despite its appeal, there is an ongoing debate on how QST should be used in the clinic. While larger university hospitals have the resources to perform multiple QST modalities on patients, many clinical settings are limited to use of one or two QST modalities to measure somatosensory function, due to the expensive equipment and the highly specialised training required for QST implementation.^23 50^ Notably, in our cohort, no single QST modality was able to distinguish between participants with and without CWP diagnosis, DED diagnosis or IBS diagnosis. This was true with both Mann-Whitney U tests and mixed effects logistic regressions, adjusted for twin relatedness, age and BMI category. We also found no difference between QST scores in participants with prevalent CPS diagnoses at the time of QST measures and participants who were diagnosed with incident CPS later, suggesting that temporality between diagnosis and QST does not impact this outcome. This is in line with previous literature determining a lack of association between QST and migraine diagnosis; migraine, while not part of the genetic CPS cluster, is considered a common overlapping condition.^51 52^ In a small subset of our sample, we reported associations between presence of DED pain symptoms and heat QST modalities (HPT, HPST); however, this study also did not find significant differences in HPT or HPST between participants with and without a DED diagnosis.^53^ Thus, while the presence of certain subsets of pain symptoms may be associated with specific QST modalities, the null associations in the present study suggest that single QST modalities are unable to capture the heterogeneity of CPS phenotypes. This highlights the need for careful interpretation of existing QST data in CPS patients and clarification of the utility and limits of QST prior to clinical implementation that requires further exploration in future studies.

One of the strengths of our study is the large participant sample size who undertook QST measures. Pain thresholds for heat stimuli were determined in approximately 3000 participants, while pain thresholds for mechanical stimuli were determined in approximately 380 participants. QST studies are typically conducted in patient cohorts with less than 100 controls. In addition, our participants were sampled from a well-characterised cohort demonstrated to resemble an age-matched, population-based British cohort.^31^ No association with CPS status was detected with minimal detectable effect sizes of 0.163–0.186 at 80.0% power (1-β) in the heat modalities and 0.421–0.456 in the mechanical modalities (online supplemental table S7). If these associations do exist, they are likely to be small.

### Individual inflammatory markers in CPS

This study is, to our knowledge, the largest analysis of IL-6, IL-8, IL-10, MCP-1 and TNF levels in participants with CPS. Selected *a priori* according to current literature, no inflammatory markers were significantly associated with CPS diagnosis in the case–control mixed effects analysis following adjustment for age, BMI category and twin relatedness; similar results were obtained in the sensitivity analysis in discordant twin pairs. This consistency of results across analyses is significant, considering the advantages of the discordant twin design—primarily the inherent matching for age, genotype (totally for MZ twins, partially for DZ twins) and most socioeconomic and environmental factors across comparison groups without additional adjustment.^54^

A recent systematic review and meta-analysis of 29 studies (N=2458) reported significant increases of TNF, IL-6, IL-8 and IL-10 in CWP/fibromyalgia patients compared with healthy controls.^55^ The component studies of this review paint a more complex picture—some reported significant increases in inflammatory marker levels, but others reported significant decreases or no significant differences. Many of the studies did not apply appropriate multiple testing corrections or adjust for age and BMI in their analyses.^55^,^59^ Our results add to previous research by addressing this limitation and suggest significant increases in levels of candidate markers in CWP patients may be attributable to the inflammation associated with age and BMI than to CWP (online supplemental table S10). A similar review in IBS has pointed to the large overlap of IL-6, IL-8, IL-10 and TNF levels between patients and healthy controls in numerous studies, despite meta-analytic reports of cytokine imbalance.^60^ Studies included in the IBS meta-analysis also did not adjust for age or BMI. Associations between candidate cytokines and DED have primarily been derived from tear samples.^27 36^ The failure to replicate these associations may be due to our analysis being conducted in blood serum samples when DED is a tear and ocular surface disease. The systemic inflammation postulated to play a role in CPS may be so low, it is not reflected in levels of individual markers. Future studies examining metabolomic pathway analyses may demonstrate inflammatory pathways are over-represented in CPS patients compared with controls.

The absence of differences in inflammatory marker levels between participants with CPS and control participants does not necessarily indicate their absolute inability to be used for phenotyping purposes. Specific subtypes of each CPS reportedly have strong associations with candidate cytokines when compared with healthy controls.^34 35^ For example, one report noted MCP-1 was not elevated in IBS patients compared with controls, but levels were significantly higher in IBS patients with metabolic syndrome than controls.^35^ Our study sample may have had an under-representation of these phenotypes and an over-representation of other phenotypes unassociated with our selected cytokines. This could potentially explain opposing concentration trends and overlapping cytokine levels seen in CPS patients when compared with healthy controls in current literature and should be further explored in future studies.

We recognise the limitations in our study. First, common dynamic QST modalities were not included in our protocol, and larger QST sample size was restricted to static heat and mechanical modalities. Compared with minimal detectable effect sizes of 0.163–0.186 at 80.0% power (1-β) in heat tests, we were only powered to detect effect sizes of 0.802–0.941 in the dynamic thermal burn tests (online supplemental table S7). This was unavoidable as a secondary data analysis. Our conclusions, therefore, draw primarily from heat and mechanical static tests; other QST results must be interpreted more cautiously. Further population studies with dynamic QST modalities and increased sample size may indicate stronger associations in CPS.

We recognise that there is a large variation in the number for participants completing each QST. As a secondary data analysis, we were unable improve sample sizes. Our sample was primarily restricted to women, with men being excluded from inflammatory marker analyses, meaning results cannot be generalised to men.

Not all participants received QST and had serum samples collected on the same day. Given the agreement between results of the main analyses and temporally restricted sensitivity analyses, we believe comparison between the QST and inflammation analyses are viable.

Perhaps our greatest limitation is that participants with common painful conditions, beyond CPS (ie, osteoarthritis), were not excluded from the analysis. While CPS case status was determined through validated diagnostic questionnaires, comorbid pain conditions have the potential to influence pain sensitivity and inflammation levels.

Our findings have several implications. We found no association between single QST and CPS in a large cross-sectional analysis of over 3000 adult volunteers. Despite using a highly sensitive proteomic assay from Olink, we did not detect association between individual circulating inflammatory markers and CPS. The lack of associations demonstrates limitations of both approaches in CPS.

## supplementary material

10.1136/bmjopen-2024-085814 online supplemental file 1

## Data Availability

Data are available upon reasonable request at https://twinsuk.ac.uk/resources-for-researchers/access-our-data/.
