# Supplementary material for: Quantitative sensory testing and chronic pain syndromes: a cross-sectional study from TwinsUK
Source: BMJ Open. 2024 Sep 3;14(9):e085814. doi: 10.1136/bmjopen-2024-085814 (PMC11407192; doi:10.1136/bmjopen-2024-085814)
Supplement: online supplemental file 1 [file bmjopen-14-9-s001.pdf]

Supporting Information

S1 QST Protocols

The cold pressor tests are utilized to measure time exposed to cold to first perceived pain and perceived intolerable pain. Participants submerged a whole hand into an ice-bath cooled to 2°C. If this was intolerable, fingers up to the knuckles were submerged. Participants indicated when they first felt pain and to remove their hand when the pain became intolerable. Times from submersion to perception of pain was recorded as CPT. Time from submersion to pain intolerance was recorded as CIT. If a participant was able to keep their hand submerged for 2 min, they removed their hand to prevent nerve damage. Shorter submersion times indicated higher pain sensitivity and lower pain tolerance.

HPT and HPST were used to measure heat pain sensitivity and tolerance respectively. Participants rested their arm on a table in front of them while in a seated position. Following inspection for possible confounding factors (e.g. cuts, bruises), a 25 x 50 mm probe connected to a Modular Sensory Analyzer (Somedic) was secured onto the volar surface of the forearm [1]. To measure HPT, the probe was slowly heated from an adaptation temperature of 32°C at a rate of 0.5°C/s until the participant stopped the test at the onset of perceived pain by pressing a button. Pain onset temperature (HPT) was recorded, and the probe temperature returned to 32°C. Probe temperature did not exceed 50°C to prevent thermal burning. A lower HPT indicates higher pain sensitivity. All participants were given standardized instructions and a practice run of the HPT experiment prior to testing.

To measure HPST, the probe was removed from the starting arm and placed at the same position on the opposite arm. From 32°C the probe was heated at a rate of 1°C/s until the participant pressed a button to indicate change in stimulus perception from painful to unbearable. The temperature at this point was logged as HPST, and the probe was quickly

returned to the adaptation temperature. If the test was not stopped before reaching 50°C, the probe temperature was automatically returned to 32°C to prevent thermal burning. A higher HPST indicates higher pain tolerance. All participants were given standardized instructions. There was no practice run when assessing HPST.

MDT was measured using a standardized set of modified von Frey hairs (Optihair2-Set, Marstock Nervtest, Germany) to assess sensitivity to mechanical force. Filaments were applied to the forearm. If participants were able to detect the first presentation, a 32 mN von Frey hair, lighter force hairs were applied. The force of the last von Frey filament the participant could sense upon application was recorded as MDT. Lower MDT scores reflect higher mechanical force sensitivity. MPT was measured using custom-made weighted pinprick mechanical stimulators to assess pinprick hypoalgesia, a decreased sensitivity to painful stimuli. Pinpricks were applied to the participant's forearm in ascending force order from 8 mN. MPT identifies the first force at which a blunt pinprick is perceived as sharp. A higher MPT indicates higher hypoalgesia. Both MDT and MPT tests were repeated a total of five times, and the final scores for each test were the geometric mean values of the trials.

The thermal burn protocol has previously been described and includes a number of measures of sensitization following a thermal burn, including: pain during burn induction, skin flare extent, punctate hyperalgesia, and thermal hyperalgesia [2]. A 32 mm^2 probe connected to a servo-controlled Peltier device (TSA-II, Medoc, Israel) was secured onto the volar surface of the right forearm with a fabric-covered band, approximately equidistant between the elbow and wrist. A mild thermal burn injury was induced by heating the probe from 32°C to 45°C at a rate of 0.5°C/s and then maintained at 45°C for 330 s. Pain intensity at 0 s, 120 s, and 210 s were recorded on a 100 mm visual analogue scale (VAS) measured to 1 mm accuracy. 0 was defined as 'no pain' and 100 as 'the worst pain you can imagine.' These pain ratings were added and recorded to provide a total rating of pain intensity during the burn protocol (out of 300).

At the end of the thermal burn procedure, the burn site was marked on the skin according to an acetate template. Skin flare (reddening of unheated skin due to the primary burn site) was measured at each spoke to the nearest 0.5 cm and used to calculate a total area of flare extent. Larger flare area reflected greater inflammation in response to thermal burn. Punctate hyperalgesia, an increased sensation of pain following a perpendicular application of a mechanical force to the skin, was assessed using a 128 mN pinprick stimulus. The stimulus was applied to the outermost point of each spoke to serve as a reference point for normal sensation for the participant; the stimulus was then applied to each dot along the spoke inward towards the primary burn area. If the stimulus was perceived to be too painful, a smaller pinprick stimulus (i.e., 64 mN) was used. The pressure was maintained for approximately 1 s at each point and stopped once the participant reported a change in sensation from ‘a prodding sensation’ to a ‘sharp pricking.’ The distance of this point from the outermost point of the spoke was recorded and repeated across all spokes to calculate an area of increased pain sensation (punctate hyperalgesia). 15 min after the thermal burn induction, the time of peak response, flare extent and punctate hyperalgesia were measured and calculated a second time using the same technique. The average of the initial and secondary measurements of flare extent and punctate hyperalgesia were used in analysis.

Thermal hyperalgesia, an increased sensation of pain following the application of a thermal stimulus to the skin, was assessed by re-measuring HPT on the same arm as first measurement. Thermal hyperalgesia was calculated as the change in HPT before and after thermal burn. A negative value indicated increased pain sensitivity at a lower temperature.

For all sensory tests, participants had their eyes closed to prevent visual clues from influencing pain perception.

1. Vehof J, Kozareva D, Hysi PG, Harris J, Nessa A, Williams FK, et al. Relationship Between Dry Eye Symptoms and Pain Sensitivity. JAMA Ophthalmology. 2013;131(10):1304-8.

2. Norbury TA, MacGregor AJ, Urwin J, Spector TD, McMahon SB. Heritability of responses to painful stimuli in women: a classical twin study. Brain. 2007;130(11):3041-9.

S1 Fig. Venn diagram of QST analytical populations by CPS

(A) By CPS questionnaire completion (B) By CPS diagnosis

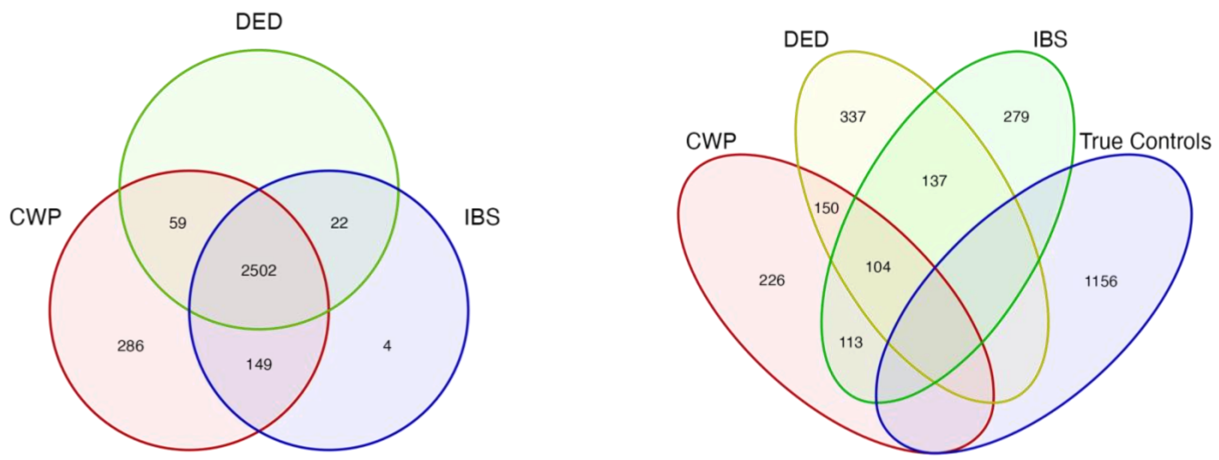

CPSs, chronic pain syndrome; CWP, chronic widespread pain; IBS, irritable bowel syndrome; DED, dry eye disease

(A) Venn diagram includes all unique participants represented in Tables 1-3 (N=3022). Each ellipse represents a CPS questionnaire completed by participants with quantitative sensory testing (QST) data and serves as an analytical group. Overlap indicates completion of multiple CPS questionnaires by participants. These participants were included in multiple analytical groups. (B) Venn diagram depicts a subset of participants represented in S1A Fig who completed all CPS questionnaires (N=2502). Each ellipse represents participants with a diagnosis of the indicated CPS or participants with no CPS (true controls). Overlap indicates multiple CPS diagnoses.

S2 Fig. Venn diagram of participants with inflammatory marker data by CPS

(A) By CPS Questionnaire Completion (B) By CPS Diagnosis

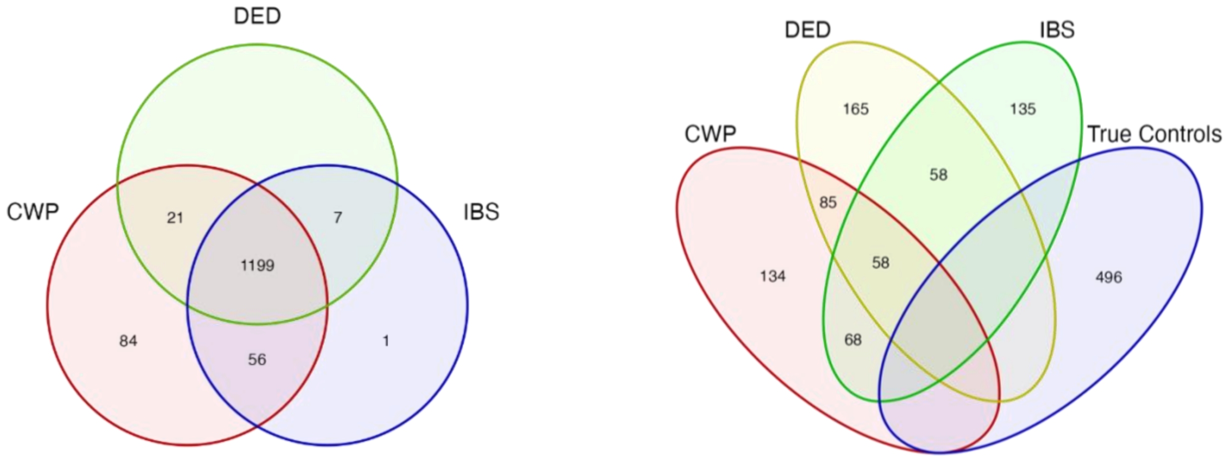

CPS, chronic pain syndromes; CWP, chronic widespread pain; IBS, irritable bowel syndrome; DED, dry eye disease

(A) Venn diagram includes all unique participants represented in S1, S3, S5 Tables (N=1368). Each ellipse represents a CPS questionnaire completed by participants with quantitative sensory testing (QST) and inflammatory marker data and serves as an analytical group. Overlap indicates completion of multiple CPS questionnaires by participants. These participants were included in multiple analytical groups. (B) Venn diagram depicts a subset of participants represented in S2A Fig who completed all CPS questionnaires (N=1199). Each ellipse represents participants with a diagnosis of the indicated CPS or participants with no CPS (true controls). Overlap indicates multiple CPS diagnoses.

S3 Fig. Flowchart of study populations

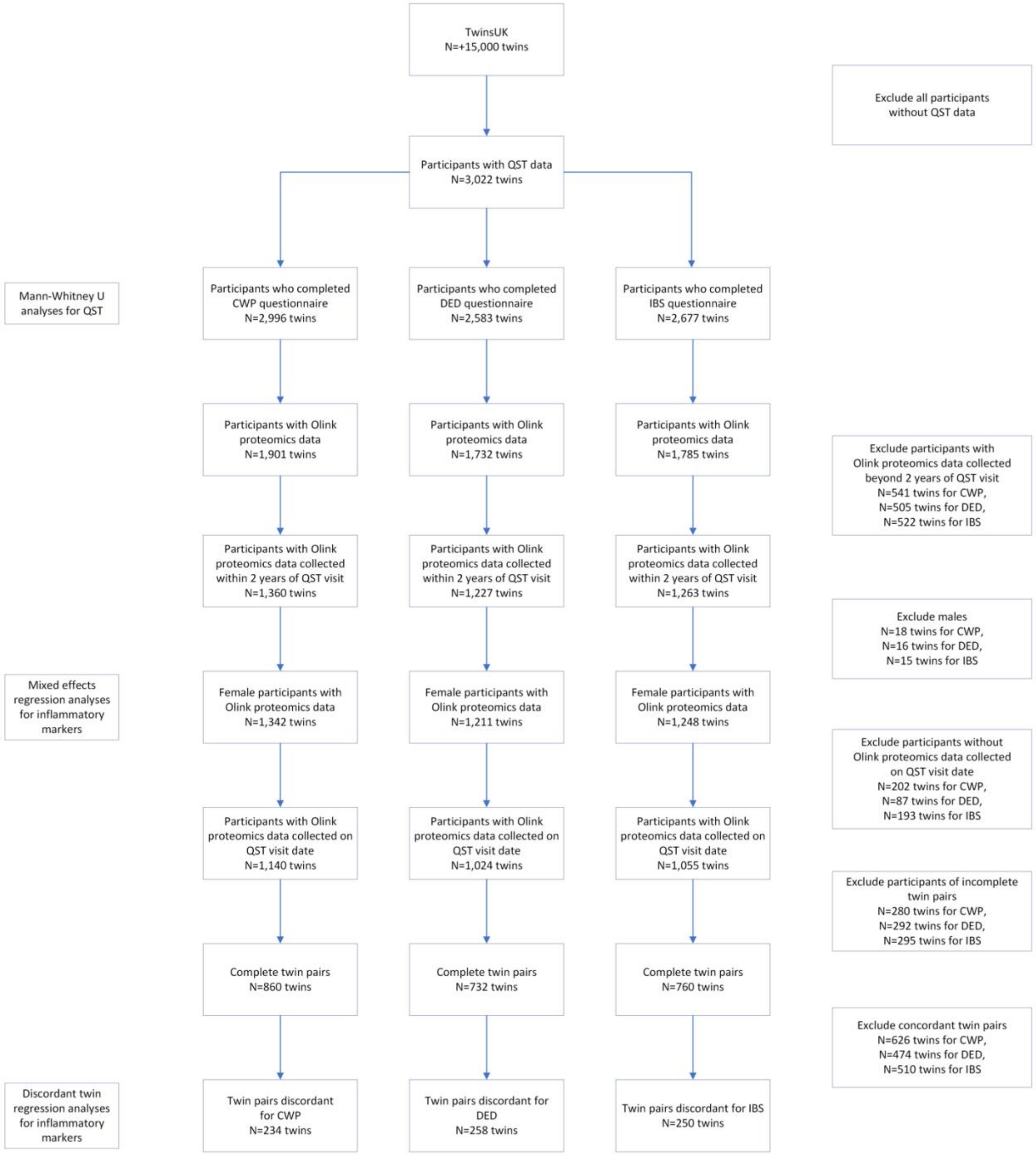

QST, quantitative sensory testing; CWP, chronic widespread pain; DED, dry eye disease; IBS, irritable bowel syndrome.

S4 Fig. Heatmap of p-values from Mann-Whitney U tests comparing QST scores in participants with a CPS and true controls.

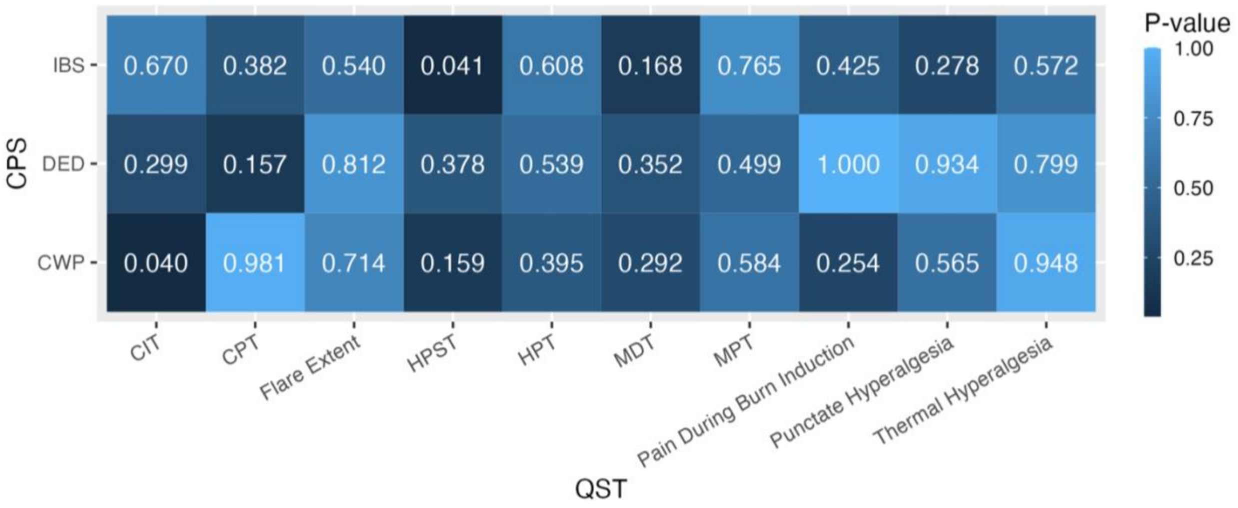

QST, quantitative sensory testing; CPS, chronic pain syndromes; CWP, chronic widespread pain; DED, dry eye disease; IBS, irritable bowel syndrome; CIT, cold intolerable threshold; CPT, cold pain threshold; HPST, heat pain supra threshold; HPT, heat pain threshold; MDT, mechanical detection threshold; MPT, mechanical pain threshold.

Each cell represents the p-value of an individual Mann-Whitney U test for the corresponding QST in the relevant CPS questionnaire population (i.e., p-value for Mann-Whitney U test comparing CIT scores in participants with CWP and true controls (participants without any CPS) = 0.040). Bonferroni-corrected p-value cutoff = 0.005.

S5 Fig. Heatmap of p-values from Mann-Whitney U tests comparing QST scores in participants with prevalent CPS and incident CPS.

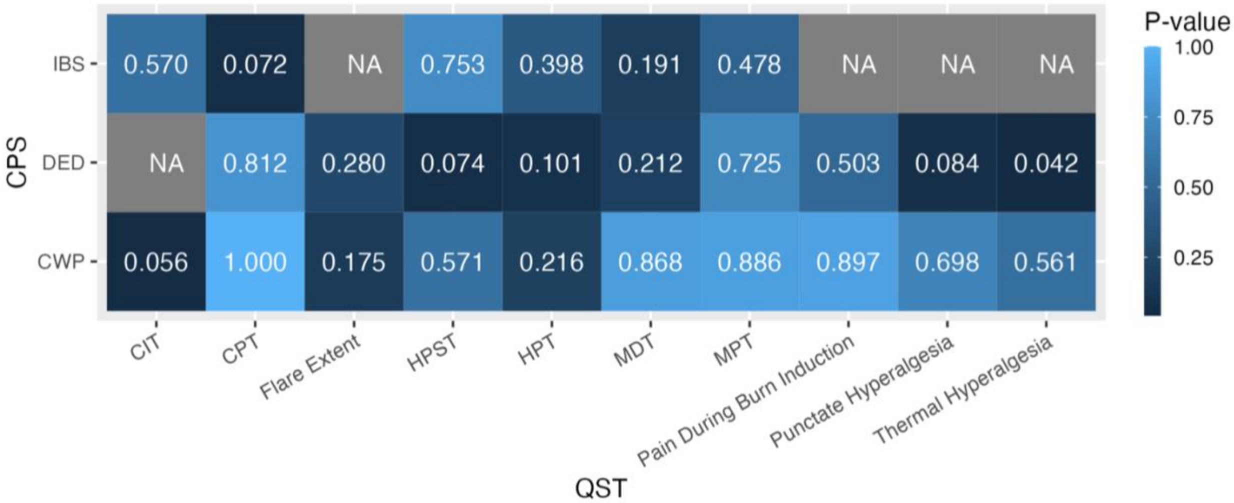

QST, quantitative sensory testing; CPS, chronic pain syndromes; CWP, chronic widespread pain; DED, dry eye disease; IBS, irritable bowel syndrome; CIT, cold intolerable threshold; CPT, cold pain threshold; HPST, heat pain supra threshold; HPT, heat pain threshold; MDT, mechanical detection threshold; MPT, mechanical pain threshold; NA, not available.

Each cell represents the p-value of an individual Mann-Whitney U test for the corresponding QST in the relevant CPS questionnaire population (i.e., p-value for Mann-Whitney U test comparing CIT scores in participants with prevalent CWP at QST visit and participants with incident CWP = 0.056). Cells marked NA did not have enough participants with the CPS of interest at date of QST for appropriate comparison. Bonferroni-corrected p-value cutoff = 0.005.

S1 Table. Characteristics of female QST participants with CWP data and inflammatory markers collected within two years of QST visit

	**Total[1] (N=1342)**	**Participants without CWP (N=973)**	**Participants with CWP (N=369)**
**Zygosity**			
Dizygotic (%)	782 (58.3)	568 (58.4)	214 (58.0)
Monozygotic (%)	552 (41.1)	398 (40.9)	154 (41.7)
Missing (%)	8 (0.6)	7 (0.7)	1 (0.3)
**Age (years)**			
Mean (SD)	60.7 (9.5)	60.1 (9.9)	62.3 (8.2)
**BMI Category**			
Underweight (%)	15 (1.1)	10 (1.0)	5 (1.4)
Healthy (%)	556 (41.4)	430 (44.2)	126 (34.1)
Overweight (%)	513 (38.2)	369 (37.9)	144 (39.0)
Obese (%)	258 (19.2)	164 (16.9)	94 (25.5)

CWP, chronic widespread pain; QST, quantitative sensory testing; SD, standard deviation; BMI, body mass index.

[1] Total female participants in TwinsUK who have completed a CWP questionnaire and at least one QST modality with inflammatory marker samples collected within 2 years of QST visit date.

S2 Table. Characteristics of female twin pairs discordant for CWP diagnosis with inflammatory markers collected on date of QST visit

	Total[1] (N=234)	Participants without CWP (N=117)	Participants with CWP (N=117)
**Zygosity**			
Dizygotic (%)	172 (73.5)	86 (73.5)	86 (73.5)
Monozygotic (%)	60 (25.6)	30 (25.6)	30 (25.6)
Missing (%)	2 (0.9)	1 (0.9)	1 (0.9)
**Age (years)**			
Mean (SD)	62.0 (9.3)	62.0 (9.3)	62.0 (9.3)
**BMI Category**			
Underweight (%)	2 (0.9)	1 (0.9)	1 (0.9)
Healthy (%)	99 (42.3)	52 (44.4)	47 (40.2)
Overweight (%)	80 (34.2)	41 (35.0)	39 (33.3)
Obese (%)	53 (22.6)	23 (19.7)	30 (25.6)

CWP, chronic widespread pain; QST, quantitative sensory testing; SD, standard deviation; BMI, body mass index.

[1] Total female twins in TwinsUK discordant with their twin for CWP diagnosis with inflammatory marker samples collected on date of QST visit.

S3 Table. Characteristics of female QST participants with DED data and inflammatory markers collected within two years of QST visit

	Total[1] (N=1211)	Participants without DED (N=843)	Participants with DED (N=368)
**Zygosity**			
Dizygotic (%)	703 (58.1)	505 (59.9)	198 (53.8)
Monozygotic (%)	502 (41.5)	332 (39.4)	170 (46.2)
Missing (%)	6 (0.5)	6 (0.7)	0 (0.0)
**Age (years)**			
Mean (SD)	60.9 (9.2)	60.5 (9.7)	61.8 (7.8)
**BMI Category**			
Underweight (%)	12 (1.0)	10 (1.2)	2 (0.5)
Healthy (%)	508 (41.9)	349 (41.4)	159 (43.2)
Overweight (%)	464 (38.3)	328 (38.9)	136 (37.0)
Obese (%)	227 (18.7)	156 (18.5)	71 (19.3)

DED, dry eye disease; QST, quantitative sensory testing; SD, standard deviation; BMI, body mass index.

[1] Total female participants in TwinsUK who have completed a DED questionnaire and at least one QST modality with inflammatory marker samples collected within 2 years of QST visit date.

S4 Table. Characteristics of female twin pairs discordant for DED diagnosis with inflammatory markers collected on date of QST visit

	**Total[1] (N=258)**	**Participants without DED (N=129)**	**Participants with DED (N=129)**
**Zygosity**			
Dizygotic (%)	164 (63.6)	82 (63.6)	82 (63.6)
Monozygotic (%)	94 (36.4)	47 (36.4)	47 (36.4)
**Age (years)**			
Mean (SD)	62.2 (8.2)	62.2 (8.2)	62.3 (8.3)
**BMI Category**			
Underweight (%)	0 (0.0)	0 (0.0)	0 (0.0)
Healthy (%)	99 (38.4)	49 (38.0)	50 (38.8)
Overweight (%)	111 (43.0)	58 (45.0)	53 (41.1)
Obese (%)	48 (18.6)	22 (17.1)	26 (20.2)

DED, dry eye disease; QST, quantitative sensory testing; SD, standard deviation; BMI, body mass index.

[1] Total female twins in TwinsUK discordant with their twin for DED diagnosis with inflammatory marker samples collected on date of QST visit.

S5 Table. Characteristics of female QST participants with IBS data and inflammatory markers collected within two years of QST visit

	Total[1] (N=1248)	Participants without IBS (N=911)	Participants with IBS (N=337)
**Zygosity**			
Dizygotic (%)	722 (57.9)	532 (58.4)	190 (56.4)
Monozygotic (%)	521 (41.7)	374 (41.1)	147 (43.6)
Missing (%)	5 (0.4)	5 (0.5)	0 (0.0)
**Age (years)**			
Mean (SD)	60.7 (9.3)	61.1 (9.0)	59.5 (9.9)
**BMI Category**			
Underweight (%)	12 (1.0)	7 (0.8)	5 (1.5)
Healthy (%)	521 (41.7)	384 (42.2)	137 (40.7)
Overweight (%)	477 (38.2)	347 (38.1)	130 (38.6)
Obese (%)	238 (19.1)	173 (19.0)	65 (19.3)

IBS, irritable bowel syndrome; QST, quantitative sensory testing; SD, standard deviation; BMI, body mass index.

[1] Total female participants in TwinsUK who have completed an IBS questionnaire and at least one QST modality with inflammatory marker samples collected within 2 years of QST visit date.

S6 Table. Characteristics of female twin pairs discordant for IBS diagnosis with inflammatory markers collected on date of QST visit

	**Total[1] (N=250)**	**Participants without IBS (N=125)**	**Participants with IBS (N=125)**
**Zygosity**			
Dizygotic (%)	156 (62.4)	78 (62.4)	78 (62.4)
Monozygotic (%)	94 (37.6)	47 (37.6)	47 (37.6)
**Age (years)**			
Mean (SD)	61.6 (8.8)	61.6 (8.8)	61.7 (8.8)
**BMI Category**			
Underweight (%)	3 (1.2)	1 (0.8)	2 (1.6)
Healthy (%)	104 (41.6)	49 (39.2)	55 (44.0)
Overweight (%)	81 (32.4)	42 (33.6)	39 (31.2)
Obese (%)	62 (24.8)	33 (26.4)	29 (23.2)

IBS, irritable bowel syndrome; QST, quantitative sensory testing; SD, standard deviation; BMI, body mass index.

[1] Total female twins in TwinsUK discordant with their twin for IBS diagnosis with inflammatory marker samples collected on date of QST visit.

S7 Table. Detectable effect sizes for Mann-Whitney U tests with 80% (1-β) power

QST	CWP	DED	IBS
CIT	0.859	0.915	1.001
CPT	0.734	0.736	0.848
Flare extent	0.823	0.802	0.939
HPST	0.186	0.178	0.186
HPT	0.164	0.163	0.165
MDT	0.432	0.423	0.456
MPT	0.429	0.421	0.455
Pain During Burn Induction	0.823	0.802	0.939
Punctate Hyperalgesia	0.832	0.810	0.941
Thermal Hyperalgesia	0.823	0.802	0.939

QST, quantitative sensory testing; CWP, chronic widespread pain; DED, dry eye disease; IBS, irritable bowel syndrome; CIT, cold intolerable threshold; CPT, cold pain threshold; HPST, heat pain supra threshold; HPT, heat pain threshold; MDT, mechanical detection threshold; MPT, mechanical pain threshold.

Each cell represents the detectable effect size of an individual two-tailed Mann-Whitney U test for the corresponding QST in the relevant CPS questionnaire population (i.e., detectable effect size for Mann-Whitney U test comparing CIT scores in participants with CWP and participants without CWP = 0.859) at 80% (1-β) power and $\alpha = 0.005$. Effect sizes were determined using G*Power 3.1 [45]. Analytic samples in which Mann-Whitney U comparison groups did not meet the unequal variances assumption are shaded red.

S8 Table. QST mixed effects logistic regression analyses

(A) CIT Univariate Analyses

	**CWP**		**DED**		**IBS**	
**Term**	**OR (95% CI)**	**P-value**	**OR (95% CI)**	**P-value**	**OR (95% CI)**	**P-value**
CIT (scaled)	0.507 (0.220, 1.168)	0.111	0.912 (0.487, 1.705)	0.772	1.276 (0.564, 2.886)	0.559

(B) CIT Multivariate Analyses

	**CWP**		**DED**		**IBS**	
**Term**	**OR (95% CI)**	**P-value**	**OR (95% CI)**	**P-value**	**OR (95% CI)**	**P-value**
CIT (scaled)	0.505 (0.202, 1.263)	0.144	1.034 (0.474, 2.253)	0.933	1.317 (0.555, 3.128)	0.532
Age (scaled)	1.482 (0.353, 6.222)	0.591	2.266 (0.495, 10.37)	0.292	1.251 (0.297, 5.280)	0.760
BMI: overweight	0.921 (0.155, 5.516)	0.931	0.097 (0.010, 0.966)	0.047	0.989 (0.151, 6.493)	0.991
BMI: obese	2.020 (0.236, 17.32	0.521	0.204 (0.021, 1.967)	0.169	1.080 (0.141, 8.259)	0.941

(C) CPT Univariate Analyses

	CWP		DED		IBS	
**Term**	**OR (95% CI)**	**P-value**	**OR (95% CI)**	**P-value**	**OR (95% CI)**	**P-value**
CPT (scaled)	1.143 (0.668, 1.956)	0.626	0.718 (0.389, 1.328)	0.291	0.803 (0.408, 1.582)	0.526

(D) CPT Multivariate Analyses

	CWP		DED		IBS	
**Term**	**OR (95% CI)**	**P-value**	**OR (95% CI)**	**P-value**	**OR (95% CI)**	**P-value**
CPT (scaled)	1.109 (0.632, 1.945)	0.718	0.848 (0.443, 1.625)	0.620	0.771 (0.375, 1.587)	0.481
Age (scaled)	1.190 (0.453, 3.125)	0.724	1.932 (0.598, 6.246)	0.271	1.348 (0.405, 4.485)	0.626
BMI: overweight	1.394 (0.398, 4.880)	0.604	0.225 (0.051, 1.004)	0.051	1.786 (0.350, 9.108)	0.485
BMI: obese	0.829 (0.193, 3.556)	0.801	0.216 (0.038, 1.242)	0.086	1.298 (0.219, 7.705)	0.774

(E) Flare Extent Univariate Analyses

	CWP		DED		IBS	
**Term**	**OR (95% CI)**	**P-value**	**OR (95% CI)**	**P-value**	**OR (95% CI)**	**P-value**
Flare Extent (scaled)	0.811 (0.419, 1.570)	0.534	0.996 (0.484, 2.049)	0.991	0.733 (0.299, 1.801)	0.499

(F) Flare Extent Multivariate Analyses

	CWP		DED		IBS	
**Term**	**OR (95% CI)**	**P-value**	**OR (95% CI)**	**P-value**	**OR (95% CI)**	**P-value**
Flare Extent (scaled)	0.839 (0.417, 1.687)	0.623	0.996 (0.456, 2.179)	0.993	0.788 (0.305, 2.035)	0.622
Age (scaled)	0.983 (0.291, 3.321)	0.978	1.646 (0.373, 7.268)	0.511	1.737 (0.328, 9.206)	0.516
BMI: overweight	2.898 (0.711, 11.81)	0.138	0.513 (0.103, 2.544)	0.414	1.692 (0.224, 12.76)	0.610
BMI: obese	1.275 (0.241, 6.758)	0.775	0.198 (0.021, 1.904)	0.161	1.079 (0.106, 10.98)	0.949

(G) HPST Univariate Analyses

	**CWP**		**DED**		**IBS**	
**Term**	**OR (95% CI)**	**P-value**	**OR (95% CI)**	**P-value**	**OR (95% CI)**	**P-value**
HPST (scaled)	0.965 (0.845, 1.102)	0.597	0.993 (0.892, 1.106)	0.902	0.863 (0.769, 0.969)	0.013

(H) HPST Multivariate Analyses

	**CWP**		**DED**		**IBS**	
**Term**	**OR (95% CI)**	**P-value**	**OR (95% CI)**	**P-value**	**OR (95% CI)**	**P-value**
HPST (scaled)	0.933 (0.818, 1.063)	0.298	0.979 (0.879, 1.090)	0.693	0.858 (0.764, 0.964)	0.010
Age (scaled)	1.898 (1.564, 2.303)	<0.001	1.275 (1.106, 1.469)	<0.001	1.066 (0.915, 1.242)	0.412
BMI: overweight	1.232 (0.920, 1.650)	0.161	0.707 (0.558, 0.897)	0.004	0.758 (0.582, 0.986)	0.039
BMI: obese	1.894 (1.327, 2.705)	<0.001	0.681 (0.504, 0.919)	0.012	0.816 (0.588, 1.132)	0.223

(I) HPT Univariate Analyses

	**CWP**		**DED**		**IBS**	
**Term**	**OR (95% CI)**	**P-value**	**OR (95% CI)**	**P-value**	**OR (95% CI)**	**P-value**
HPT (scaled)	1.039 (0.922, 1.170)	0.531	0.972 (0.880, 1.073)	0.570	0.939 (0.846, 1.043)	0.243

(J) HPT Multivariate Analyses

	**CWP**		**DED**		**IBS**	
**Term**	**OR (95% CI)**	**P-value**	**OR (95% CI)**	**P-value**	**OR (95% CI)**	**P-value**
HPT (scaled)	0.986 (0.877, 1.109)	0.820	0.948 (0.858, 1.047)	0.293	0.945 (0.850, 1.051)	0.295
Age (scaled)	1.871 (1.606, 2.179)	<0.001	1.380 (1.225, 1.555)	<0.001	0.928 (0.824, 1.045)	0.220
BMI: overweight	1.253 (0.961, 1.634)	0.095	0.726 (0.582, 0.906)	0.005	0.803 (0.632, 1.022)	0.075
BMI: obese	2.270 (1.658, 3.108)	<0.001	0.728 (0.552, 0.959)	0.024	1.001 (0.747, 1.341)	0.994

(K) MDT Univariate Analyses

	**CWP**		**DED**		**IBS**	
**Term**	**OR (95% CI)**	**P-value**	**OR (95% CI)**	**P-value**	**OR (95% CI)**	**P-value**
MDT (scaled)	1.445 (0.989, 2.110)	0.057	0.625 (0.319, 1.226)	0.171	1.029 (0.734, 1.440)	0.870

(L) MDT Multivariate Analyses

	**CWP**		**DED**		**IBS**	
**Term**	**OR (95% CI)**	**P-value**	**OR (95% CI)**	**P-value**	**OR (95% CI)**	**P-value**
MDT (scaled)	1.407 (0.963, 2.057)	0.078	0.663 (0.342, 1.286)	0.224	1.003 (0.704, 1.430)	0.985
Age (scaled)	1.636 (0.945, 2.835)	0.079	1.273 (0.823, 1.970)	0.278	1.017 (0.569, 1.818)	0.954
BMI: overweight	0.900 (0.425, 1.904)	0.782	0.534 (0.280, 1.018)	0.057	2.007 (0.861, 4.678)	0.107
BMI: obese	0.976 (0.392, 2.429)	0.959	0.573 (0.256, 1.283)	0.175	1.322 (0.475, 3.683)	0.593

(M) MPT Univariate Analyses

	CWP		DED		IBS	
**Term**	**OR (95% CI)**	**P-value**	**OR (95% CI)**	**P-value**	**OR (95% CI)**	**P-value**
MPT (scaled)	1.349 (0.982, 1.852)	0.064	0.904 (0.683, 1.197)	0.483	1.165 (0.832, 1.632)	0.374

(N) MPT Multivariate Analyses

	CWP		DED		IBS	
**Term**	**OR (95% CI)**	**P-value**	**OR (95% CI)**	**P-value**	**OR (95% CI)**	**P-value**
MPT (scaled)	1.321 (0.958, 1.821)	0.090	0.927 (0.692, 1.241)	0.609	1.115 (0.790, 1.574)	0.535
Age (scaled)	1.612 (0.946, 2.744)	0.079	1.279 (0.832, 1.965)	0.262	1.010 (0.565, 1.803)	0.974
BMI: overweight	0.935 (0.448, 1.953)	0.858	0.527 (0.278, 0.999)	0.050	1.952 (0.836, 4.560)	0.122
BMI: obese	1.127 (0.468, 2.716)	0.790	0.525 (0.240, 1.148)	0.107	1.352 (0.491, 3.722)	0.559

210 (O) Pain During Burn Induction Univariate Analyses

	CWP		DED		IBS	
**Term**	**OR (95% CI)**	**P-value**	**OR (95% CI)**	**P-value**	**OR (95% CI)**	**P-value**
Pain During Burn Induction (scaled)	0.589 (0.298, 1.166)	0.129	1.098 (0.562, 2.147)	0.784	0.728 (0.348, 1.522)	0.399

211

212 (P) Pain During Burn Induction Multivariate Analyses

	CWP		DED		IBS	
**Term**	**OR (95% CI)**	**P-value**	**OR (95% CI)**	**P-value**	**OR (95% CI)**	**P-value**
Pain During Burn Induction (scaled)	0.613 (0.321, 1.171)	0.138	1.121 (0.557, 2.259)	0.749	0.712 (0.341, 1.488)	0.366
Age (scaled)	1.106 (0.343, 3.572)	0.866	1.629 (0.393, 6.742)	0.501	2.059 (0.416, 10.180)	0.376
BMI: overweight	2.934 (0.699, 12.310)	0.141	0.520 (0.106, 2.536)	0.418	1.601 (0.224, 11.440)	0.639
BMI: obese	1.389 (0.252, 7.657)	0.706	0.196 (0.021, 1.853)	0.155	1.217 (0.126, 11.760)	0.865

213

(Q) Punctate Hyperalgesia Univariate Analyses

	CWP		DED		IBS	
**Term**	**OR (95% CI)**	**P-value**	**OR (95% CI)**	**P-value**	**OR (95% CI)**	**P-value**
Punctate Hyperalgesia (scaled)	0.831 (0.450, 1.535)	0.554	1.399 (0.613, 3.191)	0.425	0.518 (0.170, 1.576)	0.247

(R) Punctate Hyperalgesia Multivariate Analyses

	CWP		DED		IBS	
**Term**	**OR (95% CI)**	**P-value**	**OR (95% CI)**	**P-value**	**OR (95% CI)**	**P-value**
Punctate Hyperalgesia (scaled)	0.883 (0.482, 1.617)	0.687	1.383 (0.560, 3.418)	0.482	0.551 (0.183, 1.657)	0.289
Age (scaled)	1.028 (0.338, 3.129)	0.961	1.792 (0.305, 10.530)	0.519	1.885 (0.388, 9.161)	0.432
BMI: overweight	2.561 (0.636, 10.320)	0.186	0.310 (0.037, 2.615)	0.282	1.428 (0.192, 10.630)	0.728
BMI: obese	1.251 (0.251, 6.240)	0.784	0.135 (0.008, 2.393)	0.172	0.988 (0.107, 9.105)	0.992

(S) Thermal Hyperalgesia Univariate Analyses

	**CWP**		**DED**		**IBS**	
**Term**	**OR (95% CI)**	**P-value**	**OR (95% CI)**	**P-value**	**OR (95% CI)**	**P-value**
Thermal Hyperalgesia (scaled)	0.948 (0.539, 1.670)	0.854	1.033 (0.537, 1.987)	0.923	0.725 (0.339, 1.550)	0.406

(T) Thermal Hyperalgesia Multivariate Analyses

	**CWP**		**DED**		**IBS**	
**Term**	**OR (95% CI)**	**P-value**	**OR (95% CI)**	**P-value**	**OR (95% CI)**	**P-value**
Thermal Hyperalgesia (scaled)	0.867 (0.484, 1.555)	0.633	0.910 (0.439, 1.887)	0.800	0.631 (0.239, 1.663)	0.351
Age (scaled)	1.109 (0.357, 3.444)	0.859	1.666 (0.398, 6.978)	0.485	1.994 (0.277, 14.360)	0.493
BMI: overweight	3.039 (0.746, 12.370)	0.121	0.522 (0.106, 2.578)	0.425	2.442 (0.169, 35.29)	0.512
BMI: obese	1.197 (0.217, 6.591)	0.326	0.187 (0.018, 1.887)	0.155	0.815 (0.049, 13.660)	0.887

CPS, chronic pain syndromes; CWP, chronic widespread pain; DED, dry eye disease; IBS, irritable bowel syndrome; OR, odds ratio; CI, confidence interval; QST, quantitative sensory testing; CIT, cold intolerable threshold; CPT, cold pain threshold; HPST, heat pain supra threshold; HPT, heat pain threshold; MDT, mechanical detection threshold; MPT, mechanical pain threshold.

Univariate model: adjusted for Family ID (random effect); no fixed effects

Multivariate model: adjusted for Family ID (random effect), age (scaled) and BMI category (nominal) (fixed effects)

S9 Table. Conditional logistic regressions of inflammatory marker level on CPS diagnosis in discordant twin pairs

(A) Twin pairs discordant for CWP

	**Univariate Model[1]**		**Multivariate Model[2]**	
**Inflammatory Marker Level (NPX)**	**OR (95% CI)**	**P-value**	**OR (95% CI)**	**P-value**
**IL-6**	1.10 (0.75, 1.62)	0.615	1.00 (0.67, 1.49)	0.985
**IL-8**	1.74 (0.97, 3.11)	0.064	1.66 (0.93, 2.97)	0.085
**IL-10**	0.86 (0.50, 1.51)	0.607	0.90 (0.51, 1.59)	0.723
**MCP-1**	1.32 (0.72, 2.41)	0.373	1.28 (0.69, 2.35)	0.435
**TNF**	0.85 (0.46, 1.58)	0.607	0.77 (0.40, 1.46)	0.417

(B) Twin pairs discordant for DED

	**Univariate Model[1]**		**Multivariate Model[2]**	
**Inflammatory Marker Level (NPX)**	**OR (95% CI)**	**P-value**	**OR (95% CI)**	**P-value**
**IL-6**	0.80 (0.53, 1.22)	0.306	0.81 (0.54, 1.22)	0.315
**IL-8**	0.94 (0.57, 1.53)	0.791	0.95 (0.58, 1.55)	0.833
**IL-10**	0.73 (0.42, 1.25)	0.252	0.74 (0.44, 1.27)	0.280
**MCP-1**	1.05 (0.58, 1.92)	0.862	1.08 (0.60, 1.94)	0.808
**TNF**	0.83 (0.53, 1.31)	0.426	0.84 (0.54, 1.32)	0.459

(C) Twin pairs discordant for IBS

	**Univariate Model[1]**		**Multivariate Model[2]**	
**Inflammatory Marker Level (NPX)**	**OR (95% CI)**	**P-value**	**OR (95% CI)**	**P-value**
**IL-6**	0.76 (0.48, 1.21)	0.252	0.84 (0.51, 1.36)	0.472
**IL-8**	1.26 (0.77, 2.07)	0.361	1.29 (0.78, 2.14)	0.326
**IL-10**	1.09 (0.63, 1.86)	0.761	1.13 (0.66, 1.95)	0.652
**MCP-1**	1.17 (0.60, 2.29)	0.652	1.21 (0.61, 2.38)	0.589
**TNF**	1.06 (0.61, 1.83)	0.832	1.10 (0.63, 1.91)	0.735

CPS, chronic pain syndromes; CWP, chronic widespread pain; DED, dry eye disease; IBS, irritable bowel syndrome; NPX, Normalized Protein eXpression; OR, odds ratio; CI, confidence interval; IL-6, interleukin-6; IL-8, interleukin-8; IL-10, interleukin-10; MCP-1, monocyte chemoattractant protein-1; TNF, tumor necrosis factor.

[1] Strata: Family ID; Covariates: none

[2] Strata: Family ID; Covariates: BMI category (nominal)

S10 Table. Inflammatory marker mixed effects logistic regression analyses

(A) IL-6 Univariate Analyses

	**CWP**		**DED**		**IBS**	
**Term**	**OR (95% CI)**	**P-value**	**OR (95% CI)**	**P-value**	**OR (95% CI)**	**P-value**
IL-6	1.307 (1.026, 1.664)	0.02996	1.109 (0.898, 1.369)	0.33646	0.937 (0.743, 1.181)	0.57955

(B) IL-6 Multivariate Analyses

	**CWP**		**DED**		**IBS**	
**Term**	**OR (95% CI)**	**P-value**	**OR (95% CI)**	**P-value**	**OR (95% CI)**	**P-value**
IL-6	1.062 (0.818, 1.377)	0.65217	1.067 (0.847, 1.343)	0.58416	0.983 (0.767, 1.261)	0.89275
Age (scaled)	1.367 (1.104, 1.692)	0.00415	1.167 (0.983, 1.385)	0.07861	0.836 (0.698, 1.002)	0.05244
BMI: overweight	1.561 (1.045, 2.332)	0.02953	0.930 (0.669, 1.293)	0.66595	0.955 (0.669, 1.365)	0.80234
BMI: obese	2.458 (1.471, 4.107)	6E-04	1.022 (0.667, 1.566)	0.92165	0.979 (0.619, 1.548)	0.92652

(C) IL-8 Univariate Analyses

	CWP		DED		IBS	
**Term**	**OR (95% CI)**	**P-value**	**OR (95% CI)**	**P-value**	**OR (95% CI)**	**P-value**
IL-8	1.283 (0.995, 1.654)	0.05483	1.027 (0.821, 1.285)	0.81548	1.201 (0.951, 1.516)	0.12401

(D) IL-8 Multivariate Analyses

	CWP		DED		IBS	
**Term**	**OR (95% CI)**	**P-value**	**OR (95% CI)**	**P-value**	**OR (95% CI)**	**P-value**
IL-8	1.169 (0.902, 1.515)	0.23672	0.967 (0.766, 1.219)	0.77464	1.291 (1.017, 1.638)	0.03555
Age (scaled)	1.356 (1.108, 1.660)	0.00307	1.239 (1.044, 1.471)	0.01412	0.778 (0.654, 0.926)	0.00466
BMI: overweight	1.484 (1.008, 2.183)	0.04522	0.906 (0.653, 1.258)	0.55721	1.009 (0.712, 1.430)	0.95979
BMI: obese	2.390 (1.485, 3.846)	0.00033	1.043 (0.692, 1.572)	0.84043	0.995 (0.645, 1.536)	0.98268

(E) IL-10 Univariate Analyses

	CWP		DED		IBS	
**Term**	**OR (95% CI)**	**P-value**	**OR (95% CI)**	**P-value**	**OR (95% CI)**	**P-value**
IL-10	0.919 (0.690, 1.224)	0.56257	1.080 (0.851, 1.371)	0.52773	1.129 (0.876, 1.454)	0.34912

(F) IL-10 Multivariate Analyses

	CWP		DED		IBS	
**Term**	**OR (95% CI)**	**P-value**	**OR (95% CI)**	**P-value**	**OR (95% CI)**	**P-value**
IL-10	0.859 (0.643, 1.148)	0.30490	1.057 (0.829, 1.348)	0.65443	1.150 (0.891, 1.483)	0.28272
Age (scaled)	1.387 (1.135, 1.696)	0.00138	1.226 (1.035, 1.452)	0.01813	0.818 (0.691, 0.968)	0.01950
BMI: overweight	1.513 (1.027, 2.229)	0.03624	0.905 (0.652, 1.255)	0.54878	1.004 (0.710, 1.418)	0.98342
BMI: obese	2.474 (1.534, 3.989)	2E-04	1.031 (0.684, 1.554)	0.88457	0.987 (0.641, 1.519)	0.95122

(G) MCP-1 Univariate Analyses

	CWP		DED		IBS	
**Term**	**OR (95% CI)**	**P-value**	**OR (95% CI)**	**P-value**	**OR (95% CI)**	**P-value**
MCP-1	1.053 (0.757, 1.466)	0.75861	1.151 (0.861, 1.538)	0.34180	1.002 (0.742, 1.353)	0.98814

(H) MCP-1 Multivariate Analyses

	CWP		DED		IBS	
**Term**	**OR (95% CI)**	**P-value**	**OR (95% CI)**	**P-value**	**OR (95% CI)**	**P-value**
MCP-1	0.886 (0.630, 1.247)	0.48810	1.061 (0.784, 1.435)	0.70067	1.106 (0.812, 1.506)	0.52246
Age (scaled)	1.406 (1.149, 1.720)	0.00093	1.225 (1.031, 1.455)	0.02116	0.800 (0.673, 0.951)	0.01119
BMI: overweight	1.483 (1.010, 2.177)	0.04435	0.906 (0.652, 1.258)	0.55447	1.014 (0.718, 1.432)	0.93783
BMI: obese	2.417 (1.506, 3.881)	0.00026	1.037 (0.687, 1.564)	0.86293	0.999 (0.649, 1.538)	0.99777

(I) TNF Univariate Analyses

	**CWP**		**DED**		**IBS**	
**Term**	**OR (95% CI)**	**P-value**	**OR (95% CI)**	**P-value**	**OR (95% CI)**	**P-value**
TNF	1.108 (0.817, 1.503)	0.50938	1.213 (0.935, 1.573)	0.14628	1.071 (0.809, 1.419)	0.63173

(I) TNF Multivariate Analyses

	**CWP**		**DED**		**IBS**	
**Term**	**OR (95% CI)**	**P-value**	**OR (95% CI)**	**P-value**	**OR (95% CI)**	**P-value**
TNF	0.938 (0.684, 1.286)	0.69235	1.121 (0.853, 1.474)	0.41092	1.183 (0.889, 1.576)	0.24941
Age (scaled)	1.400 (1.141, 1.717)	0.00126	1.212 (1.020, 1.440)	0.02926	0.792 (0.667, 0.940)	0.00768
BMI: overweight	1.483 (1.009, 2.179)	0.04499	0.907 (0.654, 1.259)	0.56052	1.017 (0.721, 1.436)	0.92279
BMI: obese	2.416 (1.501, 3.891)	0.00028	1.026 (0.681, 1.547)	0.90162	0.989 (0.643, 1.521)	0.96151

CWP, chronic widespread pain; DED, dry eye disease; IBS, irritable bowel syndrome; NPX, Normalized Protein eXpression; OR, odds ratio; CI, confidence interval; IL-6, interleukin-6; IL-8, interleukin-8; IL-10, interleukin-10; MCP-1, monocyte chemoattractant protein-1; TNF, tumor necrosis factor.

Univariate model: adjusted for Family ID (random effect); no fixed effects

Multivariate model: adjusted for Family ID (random effect), age (scaled) and BMI category (nominal) (fixed effects)

S11 Table. Discordant twin conditional logistic regression analyses

(A) IL-6 Univariate Analyses

	**CWP**		**DED**		**IBS**	
**Term**	**OR (95% CI)**	**P-value**	**OR (95% CI)**	**P-value**	**OR (95% CI)**	**P-value**
IL-6	1.103 (0.752, 1.619)	0.61468	0.813 (0.542, 1.218)	0.31453	0.765 (0.483, 1.210)	0.25234

(B) IL-6 Multivariate Analyses

	**CWP**		**DED**		**IBS**	
**Term**	**OR (95% CI)**	**P-value**	**OR (95% CI)**	**P-value**	**OR (95% CI)**	**P-value**
IL-6	0.996 (0.667, 1.488)	0.98522	0.804 (0.530, 1.221)	0.30623	0.836 (0.514, 1.361)	0.47225
BMI: overweight	1.107 (0.589, 2.079)	0.75320	0.900 (0.456, 1.774)	0.76033	0.806 (0.403, 1.615)	0.54370
BMI: obese	2.163 (0.776, 6.028)	0.13996	1.188 (0.468, 3.011)	0.71729	0.626 (0.253, 1.549)	0.31056

(C) IL-8 Univariate Analyses

	CWP		DED		IBS	
**Term**	**OR (95% CI)**	**P-value**	**OR (95% CI)**	**P-value**	**OR (95% CI)**	**P-value**
IL-8	1.735 (0.969, 3.108)	0.06384	0.949 (0.583, 1.545)	0.83317	1.261 (0.767, 2.072)	0.36107

(D) IL-8 Multivariate Analyses

	CWP		DED		IBS	
**Term**	**OR (95% CI)**	**P-value**	**OR (95% CI)**	**P-value**	**OR (95% CI)**	**P-value**
IL-8	1.665 (0.932, 2.973)	0.08479	0.936 (0.573, 1.529)	0.79108	1.289 (0.777, 2.138)	0.32600
BMI: overweight	1.208 (0.647, 2.255)	0.55370	0.861 (0.443, 1.673)	0.65831	0.706 (0.360, 1.383)	0.30975
BMI: obese	1.856 (0.712, 4.836)	0.20591	1.249 (0.519, 3.005)	0.61926	0.614 (0.266, 1.419)	0.25391

(E) IL-10 Univariate Analyses

	CWP		DED		IBS	
**Term**	**OR (95% CI)**	**P-value**	**OR (95% CI)**	**P-value**	**OR (95% CI)**	**P-value**
IL-10	0.864 (0.495, 1.507)	0.60657	0.744 (0.436, 1.272)	0.28003	1.087 (0.635, 1.861)	0.76111

(F) IL-10 Multivariate Analyses

	CWP		DED		IBS	
**Term**	**OR (95% CI)**	**P-value**	**OR (95% CI)**	**P-value**	**OR (95% CI)**	**P-value**
IL-10	0.903 (0.512, 1.590)	0.72276	0.727 (0.421, 1.255)	0.25239	1.133 (0.658, 1.954)	0.65222
BMI: overweight	1.226 (0.656, 2.294)	0.52288	0.889 (0.456, 1.733)	0.73024	0.712 (0.363, 1.398)	0.32363
BMI: obese	2.059 (0.794, 5.341)	0.13747	1.341 (0.550, 3.271)	0.51924	0.615 (0.267, 1.419)	0.25471

(G) MCP-1 Univariate Analyses

	CWP		DED		IBS	
**Term**	**OR (95% CI)**	**P-value**	**OR (95% CI)**	**P-value**	**OR (95% CI)**	**P-value**
MCP-1	1.316 (0.719, 2.411)	0.37311	1.076 (0.596, 1.941)	0.80770	1.167 (0.596, 2.285)	0.65154

(H) MCP-1 Multivariate Analyses

	CWP		DED		IBS	
**Term**	**OR (95% CI)**	**P-value**	**OR (95% CI)**	**P-value**	**OR (95% CI)**	**P-value**
MCP-1	1.276 (0.692, 2.352)	0.43534	1.055 (0.580, 1.918)	0.86166	1.206 (0.612, 2.376)	0.58862
BMI: overweight	1.208 (0.650, 2.243)	0.55013	0.872 (0.446, 1.705)	0.68888	0.712 (0.363, 1.395)	0.32209
BMI: obese	1.972 (0.765, 5.086)	0.16001	1.253 (0.520, 3.017)	0.61538	0.620 (0.269, 1.429)	0.26198

(I) TNF Univariate Analyses

	CWP		DED		IBS	
**Term**	**OR (95% CI)**	**P-value**	**OR (95% CI)**	**P-value**	**OR (95% CI)**	**P-value**
TNF	0.850 (0.458, 1.578)	0.60719	0.843 (0.537, 1.324)	0.45883	1.061 (0.614, 1.834)	0.83225

(I) TNF Multivariate Analyses

	CWP		DED		IBS	
**Term**	**OR (95% CI)**	**P-value**	**OR (95% CI)**	**P-value**	**OR (95% CI)**	**P-value**
TNF	0.766 (0.402, 1.459)	0.41676	0.833 (0.530, 1.308)	0.42638	1.100 (0.633, 1.912)	0.73517
BMI: overweight	1.167 (0.631, 2.158)	0.62318	0.841 (0.431, 1.643)	0.61316	0.716 (0.365, 1.404)	0.33092
BMI: obese	2.155 (0.823, 5.644)	0.11787	1.233 (0.511, 2.973)	0.64137	0.619 (0.268, 1.429)	0.26138

CWP, chronic widespread pain; DED, dry eye disease; IBS, irritable bowel syndrome; NPX, Normalized Protein eXpression; OR, odds ratio; CI, confidence interval; IL-6, interleukin-6; IL-8, interleukin-8; IL-10, interleukin-10; MCP-1, monocyte chemoattractant protein-1; TNF, tumor necrosis factor.

Univariate model: adjusted for Family ID (strata); no covariates

Multivariate model: adjusted for Family ID (strata) and BMI category (nominal)
