# Supplementary material for: Quantitative sensory testing and chronic pain syndromes: a cross-sectional study from TwinsUK
Source: BMJ Open. 2024 Sep 3;14(9):e085814. doi: 10.1136/bmjopen-2024-085814 (PMC11407192; doi:10.1136/bmjopen-2024-085814)
Supplement: Supplementary file 2 [file bmjopen-2024-085814.reviewer_comments.pdf]

PEER REVIEW HISTORY

BMJ Open publishes all reviews undertaken for accepted manuscripts. Reviewers are asked to complete a checklist review form and are provided with free text boxes to elaborate on their assessment. These free text comments are reproduced below.

ARTICLE DETAILS

**TITLE (PROVISIONAL)**	Quantitative sensory testing and chronic pain syndromes: a cross-sectional study from TwinsUK
**AUTHORS**	Rhee, Amber; Granville Smith, Isabelle; Compte, Roger; Vehof, Jelle; Nessa, Ayrun; Wadge, Samuel; Freidin, Maxim; Bennett, David; Williams, Frances

VERSION 1 - REVIEW

**REVIEWER NAME**	MacDermid, Joy
**REVIEWER AFFILIATION**	University of Western Ontario, Physical Therapy
**REVIEWER CONFLICT OF INTEREST**	None.
**DATE REVIEW RETURNED**	16-Mar-2024

**GENERAL COMMENTS**	This study is impressive in volume of subjects and addresses an important topic. Having reviewed multiple large database studies like this it seems that relationships are weaker than in small well controlled single site studies. While there are a variety of reasons for this particularly with respect to QST which is high user dependent, I think the potential for noise exceeding signal should be more fully acknowledged.<br>There is a lot of data to say we found nothing so the presentation while clear is dense.<br>The study is important given it questions current beliefs but I would be reluctant to state QST and inflammation not important based on this study, and so important that a balanced view of limitations and their potential impact on associations be stated.<br><br>Strengths<br><br>1. Large twin study<br>2. Multiple QST examined as predictors; and consistent findings<br>3. Multiple outcomes including QST and inflammatory markers.<br>4. Multivariate analyses to control co-variates.<br><br>Weaknesses<br>1. Very limited description of QST protocols including what areas were tests and how tailored to pathology- if at all. Not clear if done well.<br>2. Multiple raters and sites - QST highly variable between raters – inter-rater reliability unclear and if low to moderate will dissipate correlations.<br>3. Multiple conditions that have limited expectation of sensory disturbance- if including different subgroups there should be hypothesis about which have more expectation of relationships- seems a bit of a it was there so we used it…<br>4. Sex-gender based analysis not performed and given sample size and known sex differences seems a major gap.

	5. The issue of condition-specific and sex specific relationships is not adequately considered.<br>6. Discussion of sensory disruption QST tests and pain processing QST tests not clear

**REVIEWER NAME**	Hagenbeek, Fiona
**REVIEWER AFFILIATION**	Vrije Universiteit Amsterdam
**REVIEWER CONFLICT OF INTEREST**	None
**DATE REVIEW RETURNED**	22-Apr-2024

**GENERAL COMMENTS**	In their manuscript, Rhee et al. examine the association of quantitative sensory testing modalities (QST) and inflammatory biomarkers with chronic widespread pain, dry eye disease, and irritable bowel syndrome in a large population-based cohort of predominantly female twins. This is a well-written and structured manuscript, clearly describing the research questions, providing sufficient methodological detail, and clearly highlighting the study's strengths and limitations. The authors report no significant (Bonferroni adjusted) associations between QST modalities or inflammatory markers with the three chronic pain syndromes. In the discussion, the authors make an effort to contrast their findings, especially for the inflammatory markers, with existing literature and provide thorough explanations of why their findings could differ from those reported in the literature.<br>Overall, despite and because of the lack of significant findings, I feel this manuscript makes an important addition to the literature to date. Prior to accepting the manuscript for publication, I have a few small comments/questions I would like to see addressed:<br>• There is a small typo on page 7, line 33: interleukin-6 not interlukin-6.<br>• The supplementary material includes a supplementary table 16, but this table is not mentioned in the main manuscript.<br>• The main results for exploring the association of QST modalities and chronic pain syndromes relies on Mann-Whittney U-tests. One of the basic assumptions in this test is that the groups are independent. I wonder whether the authors have considered that by also including discordant twin pairs (as evident from the supplementary tables describing the discordant twin analyses for the inflammatory markers) the independence assumption is violated? And while unlikely to change the overall conclusion, would either removing discordant pairs from these analyses or adding a correction for familial clustering (see e.g., Rosner & Grove, 1999 [https://doi.org/10.1002/(SICI)1097-0258(19990615)18:11<1387::AID-SIM126>3.0.CO;2-V]) not make more sense?<br>• When exploring the association of inflammatory markers with chronic pain syndromes the authors use a regression paradigm corrected for relatedness and as sensitivity analyses, they explore differences in inflammatory marker levels in MZ and DZ twins discordant for the chronic pain syndromes. When describing the discordant twin analyses, the authors also note that discordant twin analyses inherently adjust for (partial) genetic confounding and shared environmental factors. While they briefly mentioned correction for family relatedness in the strengths and limitations section immediately following the abstract, the advantage of the discordant twin design is insufficiently stressed in the discussion. Moreover, I wonder why the authors chose to analyze the MZ and

	DZ discordant twins together rather than adhere to the more traditional co-twin control design (see e.g., Gonggrijp et al., 2023 [https://doi.org/10.1017/thg.2023.35]), i.e., 1) population-level analyses corrected for family relatedness; 2) discordant same-sex DZ twins (controlling for shared environmental factors and partially for genetic influences); and 3) discordant MZ twins (controlling for shared environmental and genetic influences)?

VERSION 1 – AUTHOR RESPONSE

Reviewer: 1

Dr. Joy MacDermid, University of Western Ontario

Comments to the Author:

This study is impressive in volume of subjects and addresses an important topic. Having reviewed multiple large database studies like this it seems that relationships are weaker than in small well controlled single site studies. While there are a variety of reasons for this particularly with respect to QST which is high user dependent, I think the potential for noise exceeding signal should be more fully acknowledged.

We thank Dr. MacDermid for taking the time to read and comment on our manuscript. As Dr. MacDermid so correctly states, we are reporting QST measures in one of the largest samples of participants that has ever been studied and the strength of the inferences to be made from our generous dataset should not be underestimated. This is a population rather than clinical sample, which translates more appropriately to a general (or population) question regarding QST ability to predict CPS.

We do not accept that there is notable potential for noise to exceed the signal – high noise drives heritability estimates towards the null by attributing noise to environmental variance (Sun et al 2018); each of the QST examined in TwinsUK has been shown to be reproducible and have heritability estimates derived (expanded further below; Williams et al 2012). In addition, we previously reported that, in a subset of the current study population, heat QST modalities (HPT, HPST) were not significantly different between participants with and without a dry eye diagnosis (DED) by a physician, consistent with results in this study, but that those reporting pain symptoms in DED had higher pain sensitivity than those without pain symptoms (Vehof et al 2013). Along these lines, in the discussion, we do not suggest that QST is not important; rather we urge for “careful interpretation of existing QST data in CPS patients and clarification of the utility and limits of QST prior to clinical implementation” (lines 296-298). To further emphasize this point, we have added the following lines to the discussion:

(Lines 288-293): “This is in line with previous literature determining a lack of association between QST and migraine diagnosis; migraine, while not part of the genetic CPS cluster, is considered a common overlapping condition [51, 52]. In a small subset of our sample, we reported associations between presence of DED pain symptoms and heat QST modalities (HPT, HPST); however, this study also did not find significant differences in HPT or HPST between participants with and without a DED diagnosis [53].”

There is a lot of data to say we found nothing so the presentation while clear is dense.

As noted, we are motivated to publish a ‘lack of association’ finding, which we realise can be difficult. We are, however, acutely aware that such communication is a fundamental tenet of scientific progress. There are many details and descriptions of both QST and pain phenotypes that we have labored to describe in the manuscript as fully as possible. In view of this comment, we have made efforts to refine the text:

(Lines 207-212): “We found no differences between the central tendencies of QST scores in participants with and without CWP for all ten QST modalities (Fig 1). Mann-Whitney U test p-values ranged from 0.076 to 0.874 with a Bonferroni threshold of p=0.005. This finding was repeated in analyses comparing QST scores in participants with and without DED and analyses comparing QST scores in participants with and without IBS. Mann-Whitney U test p-values in these CPS ranged from 0.135 to 0.994 and 0.077 to 0.773 respectively.

(Lines 216-219): “Sensitivity analyses comparing QST scores of participants with CWP and true controls were consistent with main analyses and not statistically significant (S12 Fig). Comparisons of QST scores in Differences in central tendences of QST scores between participants with prevalent CWP and participants with incident CWP were also not statistically significant (S13 Fig).”

The study is important given it questions current beliefs but I would be reluctant to state QST and inflammation not important based on this study, and so important that a balanced view of limitations and their potential impact on associations be stated.

We thank Dr. MacDermid for recognizing the importance of our manuscript. We do not believe the limitations of the study design would change the overall conclusion of this study and its contribution to the existing literature (expanded further below), but we also do not intend for readers to conclude that QST and inflammation are not important based on this study. To further emphasize this, we have added the following lines to the discussion:

(Lines 291-299): “In a small subset of our sample, we reported associations between presence of DED pain symptoms and heat QST modalities (HPT, HPST); however, this study also did not find significant differences in HPT or HPST between participants with and without a DED diagnosis [53]. Thus, while the presence of certain subsets of pain symptoms may be associated with specific QST modalities, the null associations in the present study suggest that single QST modalities are unable to capture the heterogeneity of CPS phenotypes. This highlights the need for careful interpretation of existing QST data in CPS patients and clarification of the utility and limits of QST prior to clinical implementation that requires further exploration in future studies.”

Strengths

1. Large twin study

2. Multiple QST examined as predictors; and consistent findings

3. Multiple outcomes including QST and inflammatory markers.

4. Multivariate analyses to control co-variates.

Thank you for your encouraging comments.

Weaknesses

1. Very limited description of QST protocols including what areas were tests and how tailored to pathology- if at all. Not clear if done well.

We are grateful to Dr. MacDermid for indicating that we have not sufficiently described QST protocols in the manuscript. We wonder if the Reviewer had the opportunity to see Supplemental Material 1 "QST Protocols," which presents the QST protocols in detail. The document outlines areas tested, equipment used, and relevant calculations employed to measure each QST modality. We have added the following sentences in the Methods to better direct readers to the full QST protocols. The descriptions of the protocols are lengthy and, we believe, more appropriate for the Supplementary section. At TwinsUK, we have a very high standard of data collection and have adhered to the QST collection protocols for both researcher training and data collection extremely closely as clarified in the Supplementary descriptions (Norbury et al 2007, Williams et al 2012). As mentioned above, these QST have had heritability estimates derived within our study population and investigated in multiple studies previous (Norbury et al 2007, Williams et al 2012). Reliability of measurements in TwinsUK have also been calculated in a time-and investigator-independent manner, as expanded in the next point (Norbury et al 2007, Williams et al 2012).

(Lines 59-60): "Detailed descriptions of QST protocols can be found in Supplementary 1 (S1)."

2. Multiple raters and sites - QST highly variable between raters – inter-rater reliability unclear and if low to moderate will dissipate correlations.

We agree that inter-rater variability and sites is a concern with QST data. Performing QST measurements in over 3,000 participants, we recognize there is room for human error; we noted the importance of standardization for accurate measurements in the Methods (lines 60-62). For this reason, we strived to minimize potential human error with specialized training of nurses and research assistants. All data was collected at the same site during routine TwinsUK visits. We cited Norbury et al 2007 and Williams et al 2012, where we formally assessed the reliability of QST measurements in TwinsUK. The thermal burn protocol was performed on 10 individuals by two different investigators, two weeks apart (Norbury et al 2007). Reliability for all modalities ranged from 0.34 to 0.91 in a rater-independent manner, with majority of the estimates 0.5 or higher (Norbury et al 2007, Williams et al 2012). HPT heritability was also replicated in an independent cohort of TwinsUK twins (Norbury et al 2007). We have edited the following section to clarify and highlight this information.

(Lines 56-64): "QST was administered at a single site during standard TwinsUK visits. Protocols were established in TwinsUK in collaboration with the Stephen McMahon lab, King's College London under the auspices of Prof D Bennett (co-author, now at Oxford) [32]. Detailed descriptions of QST protocols can be found in Supplementary 1 (S1). A high degree of standardization is necessary to perform QST accurately; to achieve this, nurses and research assistants underwent considerable training. Heritability and reliability of QST measures in this particular population have been formally assessed and reported previously, with heritability and inter-rater reliability estimates for each modality ranging from 0.29 to 0.55 and 0.34 to 0.91 respectively [30, 32]."

3. Multiple conditions that have limited expectation of sensory disturbance- if including different subgroups there should be hypothesis about which have more expectation of relationships- seems a bit of a it was there so we used it…

We are uncertain what the Reviewer means but agree that sensory disturbance is more commonly seen in conditions such as diabetic neuropathy, HIV neuropathy, and multiple sclerosis. The TwinsUK cohort does not typically have twin volunteers with these conditions. Our aim in this study was to investigate common conditions of high prevalence in the general population. An increasing number of

clinical studies have examined the use of QST as tools for pain assessment in these prevalent CPS (Cruz-Almeida et al 2014). These studies, as Dr. MacDermid has noted, are often small and well-controlled (Cruz-Almeida et al 2014). Our participants, by contrast, are population-based and community-dwelling. We did not expect significant differences between different subgroups because of the conditions' symptom and genetic overlap of CPS (further addressed below).

4. Sex-gender based analysis not performed and given sample size and known sex differences seems a major gap.

We thank Dr. MacDermid for this comment. We did not address sex differences in our study because it is not possible in this cohort. Due to the historic female makeup of the TwinsUK cohort, our sample, while large, is heavily predominantly female—approximately 95% for all analytic groups. We have added these details to the abstract to highlight this.

(Lines 18-19): "Results In N=3,032 twins (95.8% female), no association was identified between individual QST modalities and CPS diagnoses (CWP, DED, and IBS)."

As noted in the limitations section of the discussion (lines 357-359), our results cannot be extrapolated to males. It is important to note, however, CPS are consistently demonstrated to have higher prevalence in women than men (Falasinnu et al 2022, Paulsen et al 2014).

We incorrectly indicated the study as retrospective in the original manuscript submission. The data itself was collected prospectively. We have corrected the language in the appropriate places to indicate that the analysis was a secondary data analysis, not retrospective.

(Lines 352): "This was unavoidable as a secondary data analysis due to the retrospective nature of our available data"

(Lines 356-357): "As a secondary data analysis Given the retrospective nature of our study, we were unable improve sample sizes."

5. The issue of condition-specific and sex specific relationships is not adequately considered.

We thank Dr. MacDermid for this comment. Due to symptom and genetic overlap of CPS, we expected associations between each condition and QST to be similar and suggest consistency across conditions in a recognized cluster of syndromes. This would be in line with current literature suggesting that the different CPS are diagnostic peripheral manifestations of a common central mechanism, rather than separate mechanisms presenting each condition separately (Williams 2018). As stated above, we could not consider sex specific relationships due to the nature of our cohort, which is predominantly female, and noted results cannot be extrapolated to males for this reason (lines 357-359).

6. Discussion of sensory disruption QST tests and pain processing QST tests not clear

We thank Dr. MacDermid for this comment. As alluded to in the limitations section of the Discussion (lines 348-350), our data was restricted to primarily static QST parameters, measuring sensory disruption. Only the QST examined as part of the thermal burn protocol could be considered as assessments of pain processing. The sample sizes for these modalities were small (~n=100) in comparison to the overall cohort (~n=3000). Our conclusions therefore draw primarily from the heat

and mechanical sensory disruption tests; pain processing QST results must be interpreted more cautiously. We have added to the Discussion to highlight this.

(Lines 348-355) “Firstly, common dynamic QST modalities were not included in our protocol, and larger QST sample size was restricted to static heat and mechanical modalities. Compared to minimal detectable effect sizes of 0.156-0.198 at 80.0% power (1-□) in heat tests, we were only powered to detect effect sizes of 0.802-0.941 in the dynamic thermal burn tests (S11 Table). This was unavoidable as a secondary data analysis. Our conclusions therefore draw primarily from heat and mechanical static tests; other QST results must be interpreted more cautiously. Further population studies with dynamic QST modalities and increased sample size may indicate stronger associations in CPS.“

Reviewer: 2

Dr. Fiona Hagenbeek, Vrije Universiteit Amsterdam

Comments to the Author:

In their manuscript, Rhee et al. examine the association of quantitative sensory testing modalities (QST) and inflammatory biomarkers with chronic widespread pain, dry eye disease, and irritable bowel syndrome in a large population-based cohort of predominantly female twins. This is a well-written and structured manuscript, clearly describing the research questions, providing sufficient methodological detail, and clearly highlighting the study’s strengths and limitations. The authors report no significant (Bonferroni adjusted) associations between QST modalities or inflammatory markers with the three chronic pain syndromes. In the discussion, the authors make an effort to contrast their findings, especially for the inflammatory markers, with existing literature and provide thorough explanations of why their findings could differ from those reported in the literature.

Overall, despite and because of the lack of significant findings, I feel this manuscript makes an important addition to the literature to date.

We express our thanks to Dr. Hagenbeek for taking the time to read our manuscript. We are encouraged by these positive comments regarding the writing style and clear descriptions within the manuscript. It is heartening to read the opinion that our findings make an important addition to the current literature.

Prior to accepting the manuscript for publication, I have a few small comments/questions I would like to see addressed:

• There is a small typo on page 7, line 33: interleukin-6 not interlukin-6.

We are grateful to Dr. Hagenbeek for picking up on this error. We have made the appropriate correction (line 67).

(Lines 66-68): “Five ‘candidate’ inflammatory markers were compiled a priori as exposure variables for secondary analysis: interleukin-6 (IL-6), IL-8, IL-10, monocyte chemoattractant protein-1 (MCP-1), and tumor necrosis factor (TNF)”

• The supplementary material includes a supplementary table 16, but this table is not mentioned in the main manuscript.

We have added a sentence indicating the contents of supplementary table 16 (now supplementary table 17) (lines 269-270).

(Lines 269-270): “Full results of inflammatory marker mixed effects regression analyses can be found in S16 Table. Discordant twin analyses can be found in S17 Table.”

• The main results for exploring the association of QST modalities and chronic pain syndromes relies on Mann-Whittney U-tests. One of the basic assumptions in this test is that the groups are independent. I wonder whether the authors have considered that by also including discordant twin pairs (as evident from the supplementary tables describing the discordant twin analyses for the inflammatory markers) the independence assumption is violated? And while unlikely to change the overall conclusion, would either removing discordant pairs from these analyses or adding a correction for familial clustering (see e.g., Rosner & Grove, 1999 [https://doi.org/10.1002/(SICI)1097-0258(19990615)18:11<1387::AID-SIM126>3.0.CO;2-V]) not make more sense?

We thank Dr. Hagenbeek for raising this point. Dr. Hagenbeek makes a good comment about the limitations of Mann-Whitney U tests in our analyses. Based on these suggestions, we have also performed mixed effects logistic regressions of QST modalities and chronic pain syndromes, adjusted for familial clustering (random effect) as well as age and BMI category (fixed effects). We found these analyses to be consistent with our Mann-Whitney U test results. These results of these regression models can now be found in S14 Table and are referred to the following sections:

(Lines 97-103): “. In consideration of family relatedness and potential confounding by age and body mass index (BMI), we repeated the main analysis using mixed effects logistic regressions of each QST modality (scaled) on CWP diagnosis (i.e., regression of HPST [scaled] on CWP diagnosis) with family ID as a random effect and age (scaled) and BMI category (nominal) as fixed effects. We utilized a BOBYQA optimization technique, using the lme4 package in R [45]. BMI categories were defined according to the Centers for Disease Control and Prevention (CDC) BMI cut-off standards [46]. All sensitivity analyses were repeated for DED and IBS.”

(Lines 219-221): “Mixed effects regression analyses of QST on CWP, adjusted for twin relatedness, age (scaled), and BMI category (nominal), were also consistent with the main Mann-Whitney U findings and failed to reach statistical significance (S14 Table).”

(Lines 283-286): “Notably, in our cohort, no single QST modality was able to distinguish between participants with and without CWP diagnosis, DED diagnosis, or IBS diagnosis. This was true with both Mann-Whitney U tests and mixed effects logistic regressions, adjusted for twin relatedness, age, and BMI category.”

• When exploring the association of inflammatory markers with chronic pain syndromes the authors use a regression paradigm corrected for relatedness and as sensitivity analyses, they explore differences in inflammatory marker levels in MZ and DZ twins discordant for the chronic pain syndromes. When describing the discordant twin analyses, the authors also note that discordant twin analyses inherently adjust for (partial) genetic confounding and shared environmental factors. While they briefly mentioned correction for family relatedness in the strengths and limitations section immediately following the abstract, the advantage of the discordant twin design is insufficiently stressed in the discussion.

We are grateful to Dr. Hagenbeek for their encouraging comments. We had previously noted the advantage of the discordant twin design in the results section (line 268) but have moved this to the discussion (lines 314-318) to improve readability and better highlight this information.

(Lines 310-318): "Selected a priori according to current literature, no inflammatory markers were significantly associated with CPS diagnosis in the case-control mixed effects analysis following adjustment for age, BMI category, and twin relatedness; similar results were obtained in the sensitivity analysis in discordant twin pairs. This consistency of results across analyses is significant, considering the advantages of the discordant twin design–primarily the inherent matching for age, genotype (totally for MZ twins, partially for DZ twins), and most socioeconomic and environmental factors across comparison groups without additional adjustment [54]."

Moreover, I wonder why the authors chose to analyze the MZ and DZ discordant twins together rather than adhere to the more traditional co-twin control design (see e.g., Gonggrijp et al., 2023 https://doi.org/10.1017/thg.2023.35, i.e., 1) population-level analyses corrected for family relatedness; 2) discordant same-sex DZ twins (controlling for shared environmental factors and partially for genetic influences); and 3) discordant MZ twins (controlling for shared environmental and genetic influences)?

We thank Dr. Hagenbeek for this comment. We recognize that MZ twins have improved matching and that analyzing the MZ and DZ discordant twins separately could maximize those strengths. However, we deemed the separate MZ and DZ twin populations to be of insufficient sample size for separate analysis. We intentionally chose to analyze the MZ and DZ discordant twins together to improve power.

We are grateful to the Reviewers and the Editor-in-Chief Adrian Aldcroft for the assistance we have received to strengthen our manuscript. We trust the additions and explanations above bring our work to the required standard for BMJ Open, and will serve as an informative, interesting and enjoyable article for your readership for many years to come.

Yours sincerely,

Amber Rhee

References:

Cruz-Almeida Y, Fillingim RB. Can Quantitative Sensory Testing Move Us Closer to Mechanism-Based Pain Management? Pain Medicine. 2014;15(1):61-72.

Falasinnu T, Nguyen T, Jiang TE, Chaichian Y, Rector A, Darnall BD, et al. The Problem of Pain in Rheumatology: Clinical Profiles Associated With Concomitant Diagnoses With Chronic Overlapping Pain Conditions. ACR Open Rheumatology. 2022;4(10):890-6.

Norbury TA, MacGregor AJ, Urwin J, Spector TD, McMahon SB. Heritability of responses to painful stimuli in women: a classical twin study. Brain. 2007;130(11):3041-9.

Paulsen AJ, Cruickshanks KJ, Fischer ME, Huang GH, Klein BE, Klein R, et al. Dry eye in the beaver dam offspring study: prevalence, risk factors, and health-related quality of life. Am J Ophthalmol. 2014;157(4):799-806.

Sun S, Zhu J, Mozaffari S, Ober C, Chen M, Zhou X. Heritability estimation and differential analysis of count data with generalized linear mixed models in genomic sequencing studies. Bioinformatics. 2018;35(3):487-96.

Vehof J, Kozareva D, Hysi PG, Harris J, Nessa A, Williams FK, et al. Relationship Between Dry Eye Symptoms and Pain Sensitivity. JAMA Ophthalmology. 2013;131(10):1304-8.

Vehof J, Zavos HMS, Lachance G, Hammond CJ, Williams FMK. Shared genetic factors underlie chronic pain syndromes. Pain. 2014;155(8):1562-8.

Williams DA. Phenotypic Features of Central Sensitization. J Appl Biobehav Res. 2018;23(2):e12135.

Williams FMK, Scollen S, Cao D, Memari Y, Hyde CL, Zhang B, et al. Genes Contributing to Pain Sensitivity in the Normal Population: An Exome Sequencing Study. PLOS Genetics. 2012;8(12):e1003095.

VERSION 2 – REVIEW

**REVIEWER NAME**	Hagenbeek, Fiona
**REVIEWER AFFILIATION**	Vrije Universiteit Amsterdam
**REVIEWER CONFLICT OF INTEREST**	20-Jun-2024
**DATE REVIEW RETURNED**	None.

**GENERAL COMMENTS**	I am satisfied with how the authors have addressed the reviewer's comments and recommend the manuscript for publication.
